# Integrated analysis of genetic, behavioral, and biochemical data implicates neural stem cell-induced changes in immunity, neurotransmission and mitochondrial function in Dementia with Lewy Body mice

**DOI:** 10.1186/s40478-017-0421-0

**Published:** 2017-03-10

**Authors:** Anita Lakatos, Natalie R. S. Goldberg, Mathew Blurton-Jones

**Affiliations:** 1 Sue and Bill Gross Stem Cell Research Center, University of California, Irvine, USA; 2 Institute for Memory Impairments and Neurological Disorders, University of California, Irvine, USA; 3 Department of Neurobiology & Behavior, University of California, Irvine, USA

**Keywords:** Synuclein, Neural stem cells, Transplantation, Dopamine, Lysosome, Autophagy, Genomics, Glutamate, Memory

## Abstract

We previously demonstrated that transplantation of murine neural stem cells (NSCs) can improve motor and cognitive function in a transgenic model of Dementia with Lewy Bodies (DLB). These benefits occurred without changes in human α-synuclein pathology and were mediated in part by stem cell-induced elevation of brain–derived neurotrophic factor (BDNF). However, instrastriatal NSC transplantation likely alters the brain microenvironment via multiple mechanisms that may synergize to promote cognitive and motor recovery. The underlying neurobiology that mediates such restoration no doubt involves numerous genes acting in concert to modulate signaling within and between host brain cells and transplanted NSCs. In order to identify functionally connected gene networks and additional mechanisms that may contribute to stem cell-induced benefits, we performed weighted gene co-expression network analysis (WGCNA) on striatal tissue isolated from NSC- and vehicle-injected wild-type and DLB mice. Combining continuous behavioral and biochemical data with genome wide expression via network analysis proved to be a powerful approach; revealing significant alterations in immune response, neurotransmission, and mitochondria function. Taken together, these data shed further light on the gene network and biological processes that underlie the therapeutic effects of NSC transplantation on α-synuclein induced cognitive and motor impairments, thereby highlighting additional therapeutic targets for synucleinopathies.

**Electronic supplementary material:**

The online version of this article (doi:10.1186/s40478-017-0421-0) contains supplementary material, which is available to authorized users.

## Introduction

For the great majority of neurodegenerative diseases, neuronal damage and loss occur long before clinical symptoms first become apparent [79, 102]. Likely for this reason therapies designed to target upstream pathological initiators of disease have thus far failed in clinical trials of symptomatic patients [30, 90] In contrast, the emerging field of stem cell transplantation may offer a potentially powerful approach to restore some aspects of brain function that have previously been lost [66]. Neural stem cells (NSCs) in particular have the potential to develop into the three principle cell-types of the brain; neurons, astrocytes, and oligodendrocytes, and can also renew themselves through asymmetrical cell division [92]. Although great progress has been made toward our understanding of NSCs and their potential application in Central Nervous System (CNS) disorders, much still needs to be determined regarding the biology of these cells and their complex interactions with endogenous host tissue and disease pathology in order to maximize therapeutic benefits [2, 15, 23, 24, 40, 41, 63, 96].

We previously examined the effects of haplotype-matched murine NSC transplantation in a transgenic model of Dementia with Lewy Bodies (DLB) that overexpresses wild-type human α-synuclein (ASO mice) [41]. Interestingly, we found that NSCs could significantly improve both motor and cognitive function 1 month after transplantation into the striata of aged ASO mice. These benefits, however, were not accompanied by any changes in Lewy body-like α-synuclein inclusions. Instead, behavioral recovery was associated with significant increases in brain–derived neurotrophic factor (BDNF), tyrosine hydroxylase activity, and glutamate type I transporter (GLT-1). Furthermore, reduction of BDNF within NSCs prevented the cognitive and motor benefits of transplantation, suggesting that neurotrophic effects of NSCs played a principal role in recovery. However, in a complementary approach, we found that viral delivery of BDNF alone only partially mimicked the effects of NSC transplantation; improving motor function but failing to significantly improve cognition [41]. Thus, we concluded that NSCs likely influence a broader set of mechanisms to affect host neuronal function and behavior. In order to identify these other potential regulatory networks involved in NSC-induced functional recovery, we have now examined whole genome gene expression in striatal samples isolated from these same mice [41].

Network analysis, a quasi-dynamic modelling of transcriptomics, offers a powerful approach to gain insight into the biological mechanisms of disease and treatment related recovery [3, 58, 70, 73, 74, 103, 112]. Combining this genomic network approach with quantitative phenotype-based analysis can in turn help to unravel the complexity of neurodegenerative diseases with considerable statistical power [45, 83]. Therefore, we implemented a systems biology approach [80] that combines quantitative phenotypes with genome wide gene expression in a network analysis to gain further insight into the mechanisms that underlie stem cell-mediated functional recovery. First, we constructed potential regulatory networks using weighted gene co-expression network analysis (WCGNA), and subsequently integrated these networks with continuous disease-related quantitative behavioral and biomarker measurements. Using this approach, we successfully identified several candidate gene networks and corresponding biological mechanisms relevant to DLB disease states and NSC engraftment. Our findings indicate that NSC transplantation robustly modifies multisystem neurotransmission, mitochondrial and lysosomal function, and immune responses in close association with improved cognitive and motor function. These results therefore greatly enhance our understanding of the mechanisms by which neural stem cell transplantation modulates neuronal and behavioral function, and point toward new disease- and recovery-associated networks that warrant further consideration.

## Materials and methods

### Animals, stem cell transplantation, and behavioral tasks

All aspects of the animal genetic background, stem cell engraftment, behavioral tasks and the biomarker biochemical assays are detailed in Goldberg et al. [41]. Briefly, all animal experiments were performed in strict accordance with the University of California, Irvine animal use regulations and the NIH guide for the Care and Use of Laboratory Animals. Age- and sex-matched transgenic mice over-expressing human wild-type α-synuclein (PDGF-β-ASO line D, ASO) and wild-type littermates maintained on a congenic C57B6/J background were examined. Hippocampal/cortical GFP-expressing mouse neural stem cells (NSCs) were microdissected from syngeneic GFP-transgenic mice at postnatal day 1, grown as adherent monolayers, and transplanted at passage 15 as previously described [15, 75]. NSCs remained on ice for the duration of the transplantation procedure and retained 89–94% viability as assessed by trypan-blue exclusion of remaining cells following transplantation [41]. Wild-type (WT) and transgenic littermates (ASO) were randomly divided into groups and received intrastriatal injections of either saline (Veh) or 100,000 haplotype-matched NSCs and WT and ASO transplants were alternated to avoid any potential confounding effect of transplantation time. The combination of genotype and treatment resulted in four experimental groups: WT-Veh, WT-NSC, ASO-Veh, ASO-NSC**.** All behavioral tasks were performed and analyzed by a researcher blinded to genotype and treatment began 30 days after NSC transplantation; and followed standard protocols to perform Novel Object Recognition (NOR), Novel Place Recognition (NPR), Rotarod, and Beam Transversal tasks [5, 6, 11, 71]. Following behavioral assessment, mice were sacrificed by transcardial perfusion with 0.01 M phosphate buffered saline and then brains were hemisected to provide tissue for histological, biochemical, and RNA analyses.

### RNA extraction and biochemical analyses

For biochemical and gene expression analyses, brains were flash frozen immediately following dissection and maintained in a semi-frozen state using dry ice chips during microdissection of the dorsal striatum and all dissections were performed within 1 min/brain. Tissue was then processed to isolate both mRNA and protein via TRIzol® using the manufacture’s guidelines (Life Technologies, Inc., Carlsbad CA). RNA quality control analyses were performed by the University of California, Irvine Genomics High-Throughput Facility at the Chao Family Comprehensive Cancer Center and included assessment of RNA concentration, 260/280 and 260/230 ratios and calculation of RNA integrity number (RIN) (Additional file 1). Five mice per group with the highest quality RNA (RIN>9) were analyzed for the current gene expression study. Protein concentrations were determined by Bradford assay and normalized samples compared by SDS-PAGE Western blot. Antibodies were used to detect mature and proBDNF (Santa Cruz Biotech #sc-546), phosphorylated Tyroxine Hydroxylase (pSer31TH, Cell Signaling, #3370), and Glial High Affinity Glutamate Transporter (GLT-1, *Slc1a2,* Abcam, #ab106289*)* as detailed in Goldberg et al. [41]*.* Relative signal intensity of grayscale images was then quantified by ImageJ software and once all values were obtained sample identification was decoded. The behavioral and biomarkers measurements described above and detailed in [41] were then used as quantitative phenotypes in the WGCNA. Additional file 2: Figure S1 summarizes the experimental design.

### Affymetrix gene array processing

All animals were sacrificed and total RNA extracted from microdissected striatum as described above. Sample purity and concentration were verified by Bioanalyzer (Agilent). All 20 RNA samples were processed on a GeneChip® Mouse Gene 2.0 ST Array (Affymetrix, Santa Clara, CA) by the UCI Genomics High-Throughput Facility following the manufacture’s guidelines. All CEL files were subjected to background correction, normalization and ‘core’ summarization using the robust multiarray analysis (RMA) algorithm implemented in Bioconductor package “oligo 1.34.2”. All probes were mapped to genes based on Bioconductor package “mogene20sttranscriptcluster.db 8.4.0”. After initial quality control (QC) analysis including RNA degradation assessment (Additional file 2: Figure S2) and clustering (Additional file 2: Figure S3), one sample was marked as an outlier and omitted from subsequent analyses. Then, array probes were filtered” for unique Entrez IDs and the most variable genes across samples by applying the interquartile range (IQR) variance filter implemented in Bioconductor package “genefilter 1.52.1. Subsequently, 50% of genes were filtered out from the original dataset leaving approximately 12,300 most variable genes for downstream analysis (detailed parameters can be found in Additional file 3). To control for potential confounding effects, all samples were adjusted for sex and litter effect by using the SampleNetwork1.07 tool [77] prior to gene network construction (Additional file 2: Figure S3.C and D).

### Weighted gene correlation network analysis (WGCNA)

WGCNA (package version 1.51) implemented in R tool (version 3.2.3) was performed on all samples that passed QC using standard methods [58]. The function “blockwiseModules” was used as described in [76] to assign each gene to a “signed” network (module) with the following parameters; softPower “20”, corType “bicor”, deepSplit “4”, minModuleSize “50”, minKMEtoStay “0”, mergeCutHeight “0.25”, “detectCutHeight “0.99995” (code for module construction can be found in Additional file 3). Then, gene expression was summarized into module eigengene (ME) as the first principal component (PC) of the entire module gene expression. Consequently, the module specific PCs were correlated by using the “bi-weight mid-correlation” (bicor) method with continuous measurements of behavioral phenotypes and biomarkers. A correlation was considered significant at *p* < 0.05 with absolute correlation >0.5. The result of analysis was visualized by “ggplot2” [49], “ggtree” [109] and “circos plot” [55]. For each gene, intramodular connectivity (module membership) often referred to as “node degree” was computed to determine genes with the most numerous connections called “hub” genes for each module [58, 59]. Gephi [8], Igraph [28], RedeR [22] tools were used to depict the relationship between hub genes in some of the modules. Potential important genes were selected by plotting the absolute values of the computed intramodular connectivity and the gene significance determined by the strength of correlation to the phenotype of interest. Genes that scored high on both scales were designated to be candidate genes for stem cell treatment related biological mechanisms.

### Gene Set enrichment analysis (GSE)

To determine whether a set of genes was differentially expressed between two conditions based on a non-parametric (Kolmogorov-Smirnov) statistical modeling, we utilized Gene Set Enrichment Analysis (GSE) [94]. We used this approach to assess whether a module as a whole was significantly down- or up-regulated in one experimental condition versus the other. Thus, each module was transformed into a geneset and tested with the stand-alone version of the GSEA tool [93]. At first, all modules were examined by contrasting ASO-NSC versus ASO-Veh conditions. Subsequent experiments tested all modules in additional 4 comparisons: ASO-NSC versus WT-Veh, WT-NSC versus WT-Veh, ASO-Veh versus WT-Veh and ASO-NSC versus WT-NSC. The results were considered significant at an FDR q-value < 0.05. (The result is summarized in Additional file 4 and GSEA parameters are included in Additional file 3). The altered modules and pathways in comparison between ASO-NSC versus WT-Veh, ASO-NSC versus WT-NSC and ASO-Veh versus ASO-NSC are summarized in Additional file 5.

### *In silico* functional annotation

Biological relevance of each module was tested by performing serial gene enrichment analyses. All tools were based on either hypergeometric test, Fisher’s exact test or a combined score test. At first, we identified modules with cell type specific expression patterns by using the Specific Expression Analysis (SEA) online tool [108]. To determine whether modules corresponded to particular subcellular components, we mined the subcellular organelle database OrganelleDB [105]. We also assed the exosomal content of each module with the FunRich tool [81], exploiting the Extracellular Vesicles database [52]. Next, we performed gene ontology and pathway analysis using a web based tool, Enrichr [56], as well as ClueGo and CluePedia [14] implemented in Cytoscape and supplemented with enrichment analysis in WGCNA. Complementary to these analyses, our functional interpretation of gene modules exploited several biological databases, including the Barres RNAseq database [110] and Innate Database [18]. Additional file 2: Figure S1B outlines the network analysis and annotation workflow.

## Results

We previously demonstrated that transplantation of murine NSCs leads to significant improvements in both motor and cognitive function in a transgenic model of DLB [41]. In addition, we found that these improvements correlated with altered dopaminergic and glutamatergic signaling and were driven in part by increases in mature BDNF protein. In the current study, we aimed to build upon these findings to identify and better understand the molecular and transcriptional changes that underlie these improvements. We therefore applied a co-expression network analysis in which we combined quantitative measurements of behavioral tasks and biomarker proteins with genome wide gene expression. This approach served two main purposes: 1) to lend considerable statistical power to interpreting associations between genomic and phenotypic quantitative measurements; and 2) identify tightly correlated gene networks that may reveal cell and tissue specific biological mechanisms through co-expression analysis.

### WGCNA analysis reveals 11 gene modules associated with phenotypic traits

**Fig. 1:**
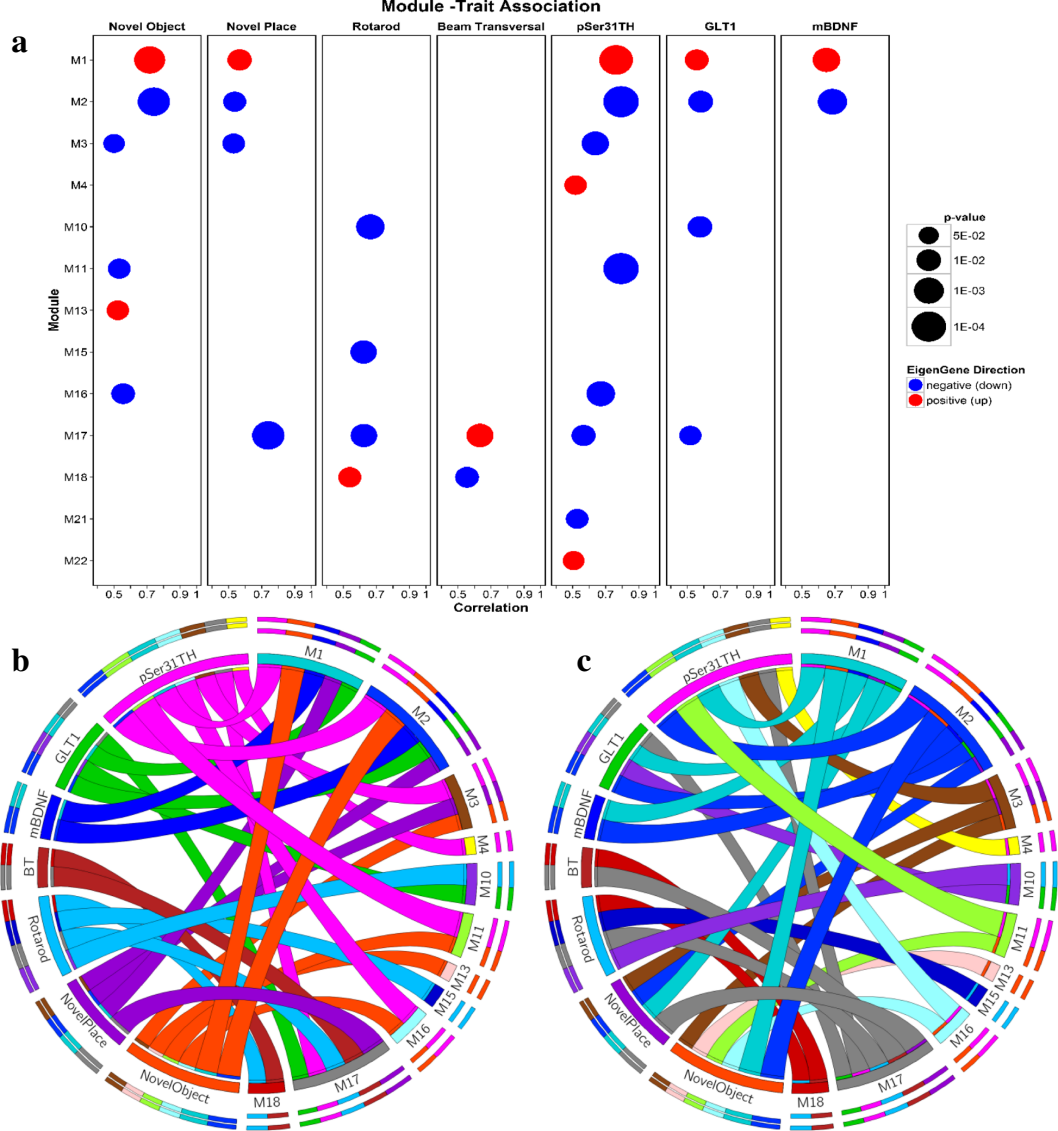
Module-Trait Association. **a** All 31 significant associations between 13 modules and 7 phenotypes are depicted as determined by bi-weight midcorrelation (bicor) between the first principal component (or EigenGene, ME) of gene expression and the continuous phenotypes (*p* < 0.05, absolute value of *r* > 0.5). Each *circle* describes a single significant correlation related to the module in association to a phenotype. The size of the circle corresponds to the significance of *p*-value. Circle color signifies the direction of the correlations and of MEs (*blue* = upregulated, *red* = downregulated). In both panels (**b**) and (**c**), the left hemisphere of the Circos plot represents the quantitative phenotypes in color-coded segments respectively while the right hemisphere represents the significant modules (M) displayed in color-coded segment designated by WGCNA. Each *ribbon* denotes a significant correlation between a phenotype and a module (29 significant associations). The number of the ribbons originating from each segment’s inner rim indicates the number of significant correlations between a phenotype and modules. Individual ribbon width demonstrates the strength of association calculated by bicor. Panel (**b**) demonstrates significant correlations colored by the origin of the phenotypes whereas panel (**c**) demonstrates significant correlations colored by the origin of the modules

We collected RNA from mouse striatum from 4 treatment groups (*n* = 5) and obtained high-throughput gene expression profiles on an Affymetrix microarray. Following microarray processing, 19 of 20 transcriptomes passed quality control and were subjected to network analysis implemented in WGCNA [58]. In an unsupervised manner, all transcriptomes were initially combined and partitioned into correlated gene sets called transcription modules. Approximately 12,000 variable genes with Entrez IDs yielded 24 sets of tightly correlated modules (Additional file 2: Figure S4). The overall gene expression of each module was then collapsed into the first principal component of gene expression variance called the “module eigengene” (ME). In a subsequent analysis, MEs were used to measure the strength of correlation between 24 modules and quantitative phenotypes (Additional file 2: Figure S5). Thirty one correlations from a total of 192 passed the statistical threshold of *p* < 0.05 and exhibited correlations less than *r* < −0.5 or larger than *r* > 0.5 with one or more behavioral tasks or biomarkers, yielding 13 significant modules of the initial 24 (Fig. 1a, Additional file 6). Since the network construction and correlational analysis were conducted in a blinded manner, we first performed gene set enrichment analysis (GSEA) to specifically address whether the modules were differentially expressed in ASO mice in response to Vehicle or NSC treatment. We found 11 of the 13 modules were significant and differentially expressed between ASO-NSC and ASO-Veh animals (Fig. 1b, Additional file 4). Table 1 summarizes the main characteristics of the significant modules. The most significant relationship (*p* < 10E-04, correlation = 0.78) was found between Module 2 and the biomarker pSer31TH which measures the phosphorylation of tyrosine hydroxylase (TH) at Serine 31, a strong indication of enzyme activity and dopamine synthesis [86]. Module 1, Module 2, and Module 17 had the largest number of significant correlations with behavioral or biomarker phenotypes (7) while biomarker pSer31TH was correlated with the highest number of modules (7) followed by performance in the Novel Object recognition task (6) (Fig. 1b, Additional file 2: Figure S5). Interestingly, most of the 11 modules especially the ones related to stem cell behavior were found similarly regulated in WT-NSC and ASO-NSC (M1, M2, M11, M13, M15, M16, M17, M18; Additonal files 7, 8, 9, 10, 11, 12, 13, 14, 15, 16, 17, 18, 19, 20) suggesting common properties of the stem cells on cellular function irrespective of disease background (Additional files 4 and 5). To determine potential mechanisms by which functional recovery occurred following NSC transplantation, we next investigated the biological significance of each module by performing *in silico* functional annotation.

**Table 1:** Summary of Significant Modules

Label	Num. of genes	Color	Eigengene (ME) in response to NSC	Associated phenotypes	Top hub gene	Gene name	Additional hub genes	Annotation
M1	1848	turquoise	up	pSer31TH, GLT1, mBDNF, NP, NO	Nat9	N-Acetyltransferase 9 (GCN5-Related, Putative)	Zfand6, C4b, Mtg2, H2-K1, Eif2ak2, Mcmbp, Herc6, Nqo1	GABAergic spiny neurons, immunity, oligodenrocyte, synapsis
M2	1499	blue	down	pSer31TH, GLT1, mBDNF, NP, NO	Ly6k	Lymphocyte Antigen 6 Complex, Locus K	Lrrc17, Spinkl, Tes, Ltbp4, Krtap13-1, Srsx, Muc6, Cpvl	cortical neurons glutamate tramsmission, immunity
M3	589	brown	down	pSer31TH, NP, NO	Elavl4	ELAV Like Neuron-Specific RNA Binding Protein 4	Nmnat2Sphkap, Fads3, Limk1, Magee1, Dner,	acetylcholinergic interneurons, synapsis
M4	300	yellow	up	pSer31TH	Itpr1	Inositol 1,4,5-Trisphosphate Receptor, Type 1	Pcdhb14, Actn2, Cacna2d3, Adcy5, Grin3	Ca regulation, synapsis
M10	65	purple	down	GLT1, Rotarod	Dnajc30	DnaJ (Hsp40) Homolog, Subfamily C,	Bloc1s1, Spg21, Tpgs1, Mtx1, Alkbh7,	mitochondria, lysosome, immunity,
M11	63	greenyellow	down	pSer31TH, NO	Olfr714	olfactory receptor 714	Kfl11, Cd101	immunity,
M13	50	salmon	up	NO	Cdhr3	cadherin-related family member 3	Spef2, Foxj1, Eno4	migration
M15	45	midnightblue	down	Rotarod	Cp	curoplasmin	Omd, Fn1, Lilrb4a	cell adhesion, immunity
M16	42	lightcyan	down	pSer31TH, NO	Olfr8	olfactory receptor 8	Slc17a3, Olfr429	immunity
M17	41	grey60	down	pSer31TH, GLT1, NO, Rotarod, BT	Snord99	small nucleolar RNA, C/D box 99	Ep400, Rpl5, Snora7a, Tmem141	mitochondria, immunity,
M18	39	lightgreen	up	Rotarod, BT	Krt25	keratin 25	Tmtc2	mitosis

*NO* Novel Object, *NP* Novel Place, *BT* Beam Transversal

### NSCs influence dopaminergic, GABAergic, and glial cell types within the striatum

**Fig. 2:**
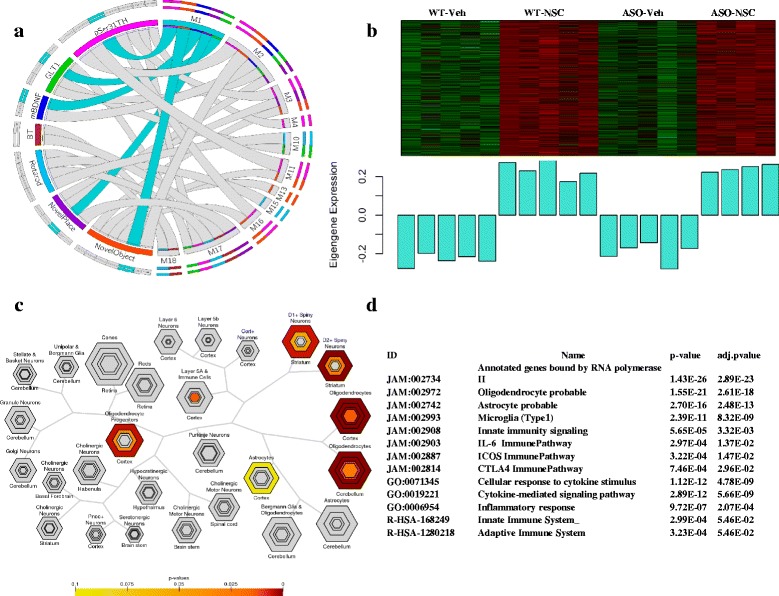
Characterization and result of the functional analysis of Module 1 (*turquoise*). **a** M1 is correlated with 5 of the 7 continuous phenotypes, including Novel Object (*p* > 6E-04, *r* = 0.72), Novel Place (*p* > 1E-02, *r* = 0.57) as well as mBDNF (*p* > 3E-03, *r* = 0.65) GLT1 (*p* > 1E-02, *r* = 0.56) and pSer31Th (*p* > 1E-04, *r* = 0.76). **b** Heatmap rows correspond to genes and columns to samples, where *red* denotes up-regulation of gene expression and *green* denotes down-regulation. The corresponding graph shows corresponding Module EigenGene (ME) expression values across the samples grouped by experimental group assignment. The ME represents the overall gene expression profile of the module, and has positive value for an array when a lot of the module genes are up-regulated (*red* in the heatmap) and has negative values when a lot of the module genes are down-regulated (*green* in the heatmap). The genes are largely down-regulated in WT-Veh and ASO-Veh, and up-regulated in WT-NSC and ASO-NSC. **c** Cell-type specific expression analysis (CSEA) identified candidate cell populations associated to M1. Each cell type is represented by a multilayer hexagonal shape in a single bullseye plot [108]. The size of hexagons for each cell type is scaled to the number of specific and enriched transcripts at four different stringency thresholds (pSI: 0.05, 0.01, 0.001, 0.0001). The more centered hexagon the more stringent threshold is and color coded by Fisher’s exact test *p* values as shown [108]. M1 is enriched in genes expressed in oligodendrocyte, spiny GABAergic neurons and less significantly in astrocytes. For example, striatal D1+ spiny neurons have many unique transcripts (large fourth hexagon at pSI < 0.05), while cortical oligodendrocytes have many unique transcripts at several stringency level (pSI < 0.05, pSI < 0.01, pSI < 0.001). **d** Representative functional annotation terms of M1 (all functional annotations can be found in Additional files 7, 8, 9 and 10)

Module 1 (M1, turquoise) contained the greatest number of genes (1848) and showed significant positive ME correlation with Novel Object recognition (*p* < 5.6E-04), Novel Place recognition (*p* < 1.1E-02), and the biomarkers pSer31TH (*p* < 1.4E-04) and mature BDNF (mBDNF; *p* < 2.0E-03) (Fig. 2a, Additional file 2: Figure S4). The large number of genes in this module likely reflects the complexity of network changes that results from incorporating one biological system (NSCs) into another multicellular biological system (mouse CNS). Although large modules such as M1 can make it difficult to understand the precise relationship between hub and connected genes, this module appears to primarily represent NSC-induced changes in gene expression that are largely independent of α-synuclein genotype. The top hub gene in M1, *Nat9* (N-Acetyltransferase 9) has previously been associated with autoimmune functions in the peripheral system, but is also highly expressed in microglia and implicated in CNS development [29, 44, 114]. Overall this module exhibited a significant upregulation of gene expression in response to NSC treatment in both WT and ASO mice (Fig. 2b). Interestingly, M1 also included significant gene sets related to stem cell biology, identifying several biological processes that are preferentially activated during stem cell proliferation and glial cell differentiation (Additional files 7, 8, 9 and 10). It follows that this module appears to be strongly related to NSC-related cellular changes, and is less influenced by α-synuclein genotype (Fig. 2b). The cell type specific enrichment analysis of Module 1 showed a strong association with D1 and D2 GABAergic spiny neurons (Fig. 2c-d), and tissue specificity pointed toward substantia nigra and striatal regions, suggesting an influence of NSCs on the nigrostriatal system, a key region of degeneration in Parkinson’s disease and DLB. Cell type specific analysis also highlighted oligodendrocytes and astrocytes, which agrees with our prior histological findings that the majority of the engrafted NSCs differentiated into glia with approximately 26% cells expressing the astrocyte specific marker (GFAP), another ~32% expressing the oligodendroglial marker OLIG2, but only ~7% expressing the early neuronal marker doublecortin (DCX) [41].

### Influence of NSCs on immune response

In addition to revealing cell types and neurocircuitry of interest, module M1 also revealed an association with both innate and adaptive immunity. M1 was significantly enriched in pathways and gene ontology terms (GO) referring to inflammation and immunity (R-HSA-168249; R-HSA-1280218; R-HSA-1280215, JAM:003031, GO:0009615, GO:0001817, GO:0045088, GO:0060337) (Fig. 2d) as well as microglia activation (JAM:002993). Gene sets related to regulatory T lymphocytes (Treg) and cytokine suppression signaling (JAM:002887, JAM:002814, JAM:002974) as well as the transcription factor FOXO1, a master regulator of Treg cells [78], were also significantly enriched (Additional files 7, 8, 9 and 10). Besides significant Foxo1 regulated genes and the CTLA immune pathway (JAM:002814), molecular markers associated with Treg functional activity such as IL10ra [62] and Cd25 (Il2ra) [85] were also components of this module. These findings are intriguing in light of a recent report that NSC-induced activation of Tregs may play a critical role in recovery and remyelination in a model of Multiple Sclerosis [23] and growing evidence that CNS cell transplantation can modulate both innate and adaptive immunity and vice versa [39, 61, 82]. These data therefore suggest that NSC-induced processes including proliferation, glia differentiation and immune modulation, may help to create a neuroprotective environment that increases dopamine transmission and behavioral performance.

The role of exosomes in cell communication and intercellular transfer of bioactive molecules has been recognized in tissues outside the nervous system and is best established among cells of the immune system, where exosomes have been demonstrated to modulate antigen presentation and the immune response [31]. Secretion of exosomes containing regulatory factors by both NSCs and newly formed neuroglia could therefore alter microenvironment cues to modulate both immune and neuronal function [9, 27, 54]. We therefore compared the content of the modules to Vesiclepedia, a compendium of molecular data of extracellular vesicles including ectosomes, exosomes and apoptotic bodies [52]. Interestingly, M1 was associated with exosomal RNAs and proteins, as was also significantly associated with the GO term ‘external side of plasma membrane’ (GO:0009897). Taken together, M1 functional annotation revealed biological processes related to GABAergic spiny neurons, nigrostriatal dopaminergic systems, stem cell glial differentiation, immunity and exosomes.

In addition to M1, modules M11 and M16 further implicate attenuated immunity. Module 11 (M11, greenyellow) included 63 genes and demonstrated a significant negative correlation with Novel Object task (*p* < 1.9E-02) and pSer31TH (*p* < 9.7E-03) (Fig. 1, Additional file 2: Figure S5) and was significantly downregulated in response to NSCs treatment in both WT and ASO mice (Additional files 4 and 5). This module is diverse but is enriched in terms related to G-protein coupled receptor signaling (GO:0007186), oxidoreductase activity (GO:0016717), and immune and inflammatory response (GO:0006954, GO:0006955) (Additional files 7, 8, 9 and 15). Likewise, module 16 (M16, ligthcyan) consisting of 42 genes demonstrated significant negative correlation with the Novel Object task (*p* < 1.3E-02) and the pSer31TH biomarker (*p* < 1.6E-03) (Fig. 1, Additional file 2: Figure S5). ME was significantly upregulated in ASO mice but was in turn downregulated by NSCs treatment. This module is associated with a number of immunological processes including cytokine-cytokine receptor interactions, and interleukin signaling pathways (Additional files 7, 8, 9 and 18). To further confirm the role of innate immunity in stem cell-mediated gene expression changes, we examined patterns of innate immunity related gene expression (InnateDB) [18] in our dataset. Clustering revealed a sharp separation between WT and ASO mice and an overall grouping of NSC transplanted mice (Additional file 2: Figure S6). Thus, it appears that NSC transplantation dramatically alters immune function in both WT and ASO brains in a manner that correlates well with cognitive function and dopaminergic signaling.

### Both α-synuclein and NSC transplantation influence cell migration

Another module that exhibited a significant positive correlation with the Novel Object cognitive task was Module 13 (M13, salmon; *p* < 2.1E-05) (Fig. 1, Additional file 2: Figure S5) which included 50 genes and primarily implicates cell migration. This module was downregulated in ASO mice compared to WT, and upregulated in response to NSC transplantation (Additional files 4 and 5) in both WT and ASO mice and was associated with neuronal migration (hs_Lis1Pathway), axoneme assembly (GO:0035082), cytoplasmic dynein complex (GO:0005868, GO:0044782, GO:0044782) movement of cell or subcellular components (GO:0006928) and NSC migration inducing cytokines (e.g., CXCR4) (Additional files 7, 8, 9 and 16). The hub gene in M13 is *Cdhr3* (Cadherin-Related Family Member 3) which is involved in cell-cell adhesion (GO:0098609, GO:0007156) including epithelial polarity, cell-cell interactions and differentiation [48]. In addition, *Cdhr3* has been implicated in tissue morphogenesis, coordinated cell movements, and the induction and maintenance of structural and functional cell and tissue polarity [43]. This module therefore likely reflects the motility of the transplanted NSCs or host immune cells throughout the striatum and into the adjacent cortices in accordance with our previously published findings [41]. Since Module 13 expression was generally downregulated in ASO compared to WT mice, it is possible that α-synuclein pathology also inhibits cell migration.

### Altered neurotransmitter and calcium signaling

**Fig. 3:**
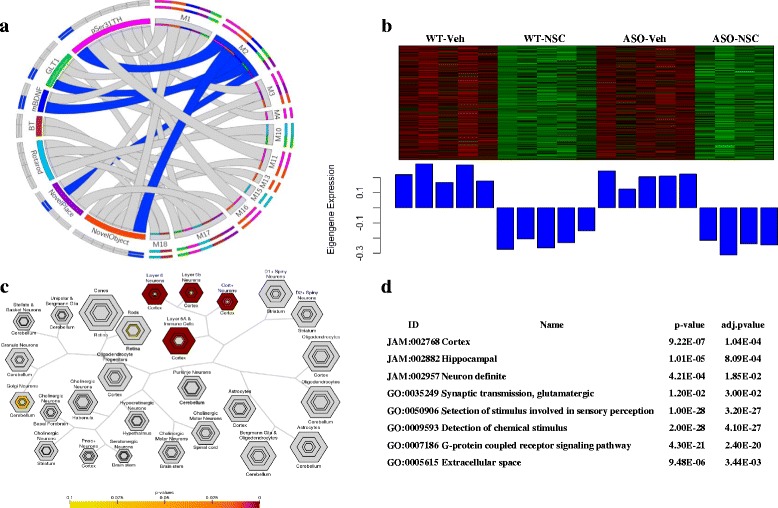
Characterization and result of the functional analysis of Module 2 (*blue*). **a** M2 is significantly associated to Novel Object (*p* > 2.8E-04, *r* = −0.74), Novel Place (*p* > 1.7E-02, *r* = −0.54) as well as mBDNF (*p* > 1.2E-03, *r* = −0.68), GLT1 (*p* > 9.0E-03, *r* = −0.58) and pSer31Th (*p* > 5.2E-05, *r* = −0.79). **b** Heatmap analysis of ME expression levels show that genes in M2 are largely increased in Veh groups and downregulated in NSC-transplanted groups. **c** Cell-type specific expression analysis (CSEA) show that M2 is enriched in genes expressed in cortical neurons and immune cells. **d** Representative functional annotation terms of M2 (all functional annotations can be found in Additional files 7, 8, 9 and 11)

Module 4 (M4, yellow) represents an especially interesting group of genes as this module was significantly downregulated in ASO mice compared to WT and NSC treatment reversed the direction of gene expression (Additional file 1) suggesting that NSC transplantation partially rescue an α-synuclein-driven perturbation of these genes. M4 consists of 300 genes and revealed a significant positive correlation with the biomarker pSer31TH (*p* < 2.3E-02) (Fig. 1, Additional file 2: Figure S5). The top hub gene in this module *Itpr1*, Inositol 1,4,5-Trisphosphate Receptor Type 1, is a member of the main endoplasmic reticulum (ER) Ca2+- release channel family that modulates intracellular calcium signaling [12]. Interestingly, mutations in *Itpr1* have been associated with another movement disorder; spinocerebellar ataxia type 15 [47]. The module significant functional annotation is related to calcium signaling (hsa04020), dopaminergic synapses (hsa04728), synaptic transmission (GO:0007268), postsynaptic density (GO:0045211, GO:0045211), locomotor behavior (GO:0007626), ion channel complex (GO:0034702) and voltage-gated cation channel activity (GO:0022843) (Additional files 7, 8, 9 and 13). The correlation of Module 4 with the pSerTH31 biomarker indicates a particular influence of both α-synuclein and NSC transplantation on striatal dopaminergic synapses. In accordance with these findings it has previously been reported that the synaptic accumulation of α-synuclein triggers the redistribution of several presynaptic proteins including SNAP-25, syntaxin-1, and synaptobrevin-2 resulting in an age-dependent reduction in dopamine release [20, 37]. It is also noteworthy that, one of the signaling events that can influence the ERK cascade which phosphorylates Ser31 of TH, is a change in free intracellular calcium concentrations [1, 26]. This module may therefore represent Calcium mediated signaling that leads to improved synaptic plasticity and phosphorylation of TH by MAPK cascades in postsynaptic terminal of dopaminergic projection neurons. Module 2 (M2, blue) also appeared to be indicative of changes in synaptic transmission and included 1499 genes that showed significant negative correlations with the Novel Object (*p* < 2.8E-04) and Novel Place tasks (*p* < 1.7E-02), as well as the biomarkers pSer31TH (*p* < 5.2E-05), mBDNF (*p* < 1.2E-03), and GLT1 (*p* < 9.0E-03) (Fig. 3a, Additional file 2: Figure S5). This module was also significantly downregulated in response to NSC transplantation in both WT and ASO mice (Fig. 3b). Interestingly, M2 is highly specific for cortical neurons (Fig. 3c) and enriched in genes related to glutamatergic synaptic transmission (Fig. 3d; GO:1900449, GO:0032281, GO:0007409), neuronal projection (GO:0043005, GO:0048812) and immune cells (GO:0002828) (Additional files 7, 8, 9 and 11). This module is also significantly correlated to the GLT-1 biomarker (*p* < 3.0E-03) (Fig. 3a), and is therefore likely related to our prior finding that NSC transplantation modulates the expression of both neuronal and astrocytic glutamate transporters; VGLUT1 and GLT-1 [41].

**Fig. 4:**
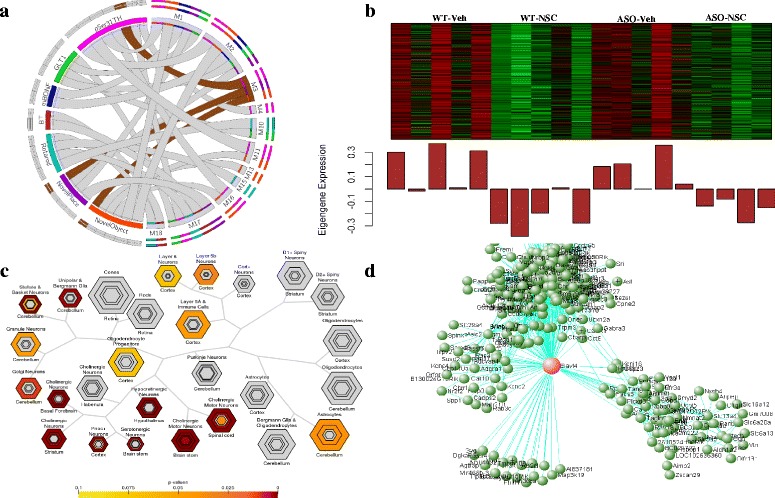
Characterization and result of the functional analysis of module M3 (*brown*). **a** M3 is significantly associated to Novel Object (*p* > 2.8E-02, *r* = −0.52), Novel Place (*p* > 1.9E-02, *r* = −0.53) and pSer31Th (*p* > 3.0E-03, *r* = −0.64). **b** Heatmap analysis of ME expression levels show that genes in M3 are largely driven by NSC transplantation, being upregulated in Veh treated groups and downregulated in NSC groups. **c** Cell-type specific expression analysis (CSEA) shows that M3 is highly enriched in genes expressed in cholinergic neurons. **d** The modular network organization of M3 depicted by VisANT [46]. Each green node represents a unique gene in M3 and the gene-gene interactions depicted in *cyan lines*. The *red* center node corresponds to the module hubgene *Elavl4* with highest intramodular connectivity holding together the entire network as a functional unit (enlarged figure: Additional file 2: Figure S7)

Clustering closely with M2 (Additional file 2: Figure S8), Module 3 (M3, brown) included 589 genes, and showed significant negative correlations with Novel Object recognition (*p* < 2.8E-02), Novel Place recognition (*p* < 1.9E-02), and pSer31TH (*p* < 3.0E-03) (Fig. 4a, Additional file 2: Figure S5). This module also showed a trend toward upregulation in ASO mice but was significantly downregulated in response to NSCs treatment in both WT and ASO mice (Fig. 4b). Interestingly, M3 was enriched in neuron specific genes (JAM:002958) indicative of striatal cholinergic interneurons and cell-type specific expression analysis (CSEA) revealed enrichment of genes expressed in cholinergic neurons (Fig. 4c). Significant GO terms for this module were related to neuronal (GO:0022008, GO:0030182, GO:0048699) and synaptic function (GO:0007268, GO:1902630) (Additional files 7, 8, 9 and 12). As cholinergic interneurons play an important role in regulating striatal output, these data could suggest that NSC-induced changes in cholinergic function may play an important role in motor or cognitive recovery. Interestingly, the top hub gene for this module is *Elavl4* (HuD/ELAV like protein 4) (Fig. 4d, Additional file 2: Figure S7). Elavl4 is implicated in neuron-specific RNA processing, dendritic morphogenesis, and learning and memory [19]. Furthermore, genetic variants in human * ELAVL4* gene have been associated with age of onset in Parkinson’s disease (PD) [35].

### α-synuclein pathology and NSC transplantation impact mitochondrial and lysosomal-related gene expression

Mitochondrial dysfunction and impairments in lysosomal function and mitophagy have been strongly implicated in the pathogenesis of Parkinson’s disease and other synucleinopathies [13, 70, 97, 107, 111]. It is therefore extremely interesting that Module 10 (M10, purple), includes 65 genes that together implicate mitochondrial and lysosomal function, oxidative stress, and apoptosis and this module exhibits a significant negative correlation with Rotarod (*p* < 1.9E-03) and the biomarker GLT1 (*p* < 9.7E-03) (Fig. 1, Additional file 2: Figure S5). Most revealing is that M10 was upregulated specifically in ASO mice, and decreased to WT expression levels by NSC transplantation. The functional annotation of this module revealed significant association with mitochondria and apoptosis, and (GO:0055114, GO:0043066, JAM:002874), and perhaps most interestingly the Parkin-Ubiquitin Proteasomal System (hs_WP2359) which has been directly linked with the lysosomal degradation of mitochondria (mitophagy) [32, 60] (Additional files 7, 8, 9 and 14). The most connected gene of this module was *Dnajc30* (DnaJ (Hsp40) Homolog, Subfamily C, Member 30, alias WBSCR18), an intron-less gene encoding a member of the DNAJ/HSP40 molecular chaperones which has been implicated in mitochondrial DNA maintenance and replication and oxidative stress [98, 99]. This gene has also been associated with Williams syndrome, a multisystem developmental disorder characterized by mild to moderate delays in cognitive development and intellectual disabilities [72]. An additional interesting hub gene in this module is *Bloc1s1*. *Bloc1s1* is a component of the BLOC-1 complex, which is involved in the biogenesis of lysosome-related organelle, suggesting a potential influence on lysosomal degradation of aggregates or dysfunctional organelles, both of which have been strongly implicated in synucleinopathies [17, 34, 50, 106]. In addition, the BLOC-1 complex is also involved in the negative regulation of aerobic respiration through mitochondrial protein lysine-acetylation [88, 89].

**Fig. 5:**
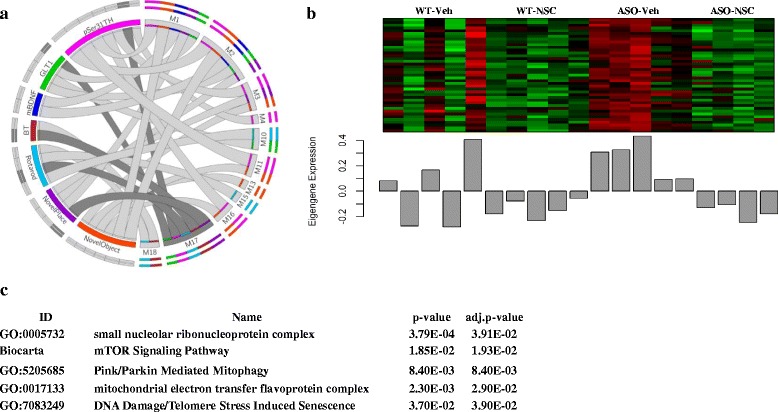
Characterization and result of the functional analysis of module M17 (*grey60*). **a** M17 is significantly associated to Novel Place (p >2.9E-04, *r* = −74), Rotarod (*p* > 4.3E-03, *r* = −0.62) and Beam Transversal (*p* > 3.6E-03, *r* = 0.63) as well as GLT1 (*p* > 2.3E-02, *r* = −0.52) and pSer31TH (*p* > 1.1E-02, *r* = −0.57). **b** The ME expression profile reveals variable expression in WT-Veh, up-regulation in ASO-Veh, and a down-regulation in WT-NSC and ASO-NSC groups. Based on the mean MEs, this module appears to be related to alpha-synuclein pathology which is rescued by NSC transplantation (all functional annotations can be found in Additional files 7, 8, 9 and 19)

Another set of genes that further implicate mitochondrial and lysosomal function and autophagy are the 41 genes that make up Module 17 (M17, grey60) which clusters closely with M10 (Additional file 2: Figure S8) and shows a significant positive correlation with Novel Place recognition (*p* < 2.9E-04) and Rotarod (*p* < 4.3E-03), a negative correlation with Beam Traversal (*p* < 3.6E-03), and positive correlation with the biomarkers pSer31TH (*p* < 1.1E-02) and GLT1 (*p* < 2.3E-02) (Fig. 5a, Additional file 2: Figure S5). This module was upregulated in ASO mice compared to WT mice, and significantly downregulated in response to NSC transplantation in both WT and ASO mice (Fig. 5b) and is enriched in small nucleolar RNAs (GO:0005732), mitochondria-associated genes (GO:0005739, GO:0098798), histone acetyl transferase activity (GO:0035267), mTOR signaling and autophagy (mTORPathway) (Fig. 5c; Additional files 7, 8, 9 and 19). Dysregulations of these processes have also been strongly implicated in synucleinopathies such as Parkinson’s disease [16, 64].

## Discussion

Due to the complex pathology of neurodegenerative disease, stem cells transplantation has been increasingly considered as a potential approach to compensate for neuronal loss, promote plasticity, and restore brain function [15, 24, 57, 63, 69, 96]. While a growing number of studies support the potential translational value of stem cell therapies for neurodegeneration, the precise mechanisms by which these cells improve behavioral function remains largely unclear [33, 63, 96]. We previously reported that transplantation of murine NSCs leads to significant improvements in both motor and cognitive behavior in a transgenic model of Dementia with Lewy Bodies (DLB) that correlated with dopaminergic and glutamatergic biomarkers and were driven at least part by increases in mature BDNF protein [41]. However, viral delivery of BDNF alone only partially mimicked the benefits of NSC transplantation, suggesting that other mechanisms are likely involved. To gain further insight into the multifactorial nature of stem cell therapy we therefore extended and complemented our previous experiment with gene expression network analysis to reveal transcriptional changes and cellular mechanisms that may underlie NSC related functional recovery in this model of DLB.

Correlational network analysis with comprehensive functional annotation provides a powerful approach to identify cell type specificity and related biochemical processes in complex systems such as NSCs transplanted into the CNS [65, 70, 104]. By comparing gene expression networks of striatal transcriptomes due to either DLB genotype or NSC transplantation, we detected several modules of highly connected genes that were significantly correlated with phenotypic changes. We report a diverse set of gene network modules that reflect changes ranging from large biological systemic changes (M1) to more focused and organelle specific changes (M17). This diversity of networks reflects the complex systemic changes that occur due to transplanting a biological system (NSCs) into another biological system (mouse CNS), and has allowed us to highlight participating clusters within larger altered networks [42, 70]. The functional annotation of genes in significant modules confirmed our previous observation that engrafted NSCs preferentially differentiate into astrocytes and oligodendrocytes. Likewise, we identified gene networks related to stem cell migration and proliferation consistent with our prior observation of robust NSC migration.

Interestingly, we also found evidence of gene enrichment associated with both pro– and anti-inflammatory state in response to NSC treatment. This anti-inflammatory activation is most likely driven by the transplant itself as a previous report has demonstrated that newly formed glial cells in the CNS elevate anti-inflammatory gene expression, counteracting pro-inflammatory cytokines and creating a more permissive environment for neuronal survival [54]. Therefore, it is possible that glial-differentiated NSCs or endogenous glial cells provide important anti-inflammatory signals following NSC transplantation. Although expression changes in genes associated with innate immunity were observed between ASO and WT mice, the α-synuclein phenotype did not have a significant effect on NSC-related gene networks. This finding, which is consistent with our prior report that NSC survival and differentiation is equivalent between ASO and WT mice, suggests that transplanted NSCs can differentiate normally regardless of α-synuclein pathology (Additional files 4 and 5).

In addition to innate inflammatory responses, our results also point toward a role for the adaptive immune system in attenuating inflammation. Emerging evidence demonstrates that certain adaptive immune cells, such as T regulatory cells (Tregs) which have a pivotal role in maintaining immunological tolerance and inhibiting inflammatory responses, can play an important role in CNS repair [4, 7, 23, 51, 54]. For example, Chen et al. demonstrated that transplantation of human embryonic stem cell-derived neural precursor cells was associated with a reduction in neuroinflammation which correlated with an increased number of CD4(+)CD25(+)FOXP3(+) regulatory T cells and sustained clinical recovery in the mouse model of multiple sclerosis (MS) [23]. Moreover, our lab also recently described a potential cross-talk between T cells and microglia in an animal model of Alzheimer’s disease further implicating the adaptive immune system in neurodegenerative disease [67]. It is therefore likely that in the ASO model, multiple cell populations contribute to either inflammatory or immunomodulatory conditions that influence behavior, and that these populations respond to and modulate one another via both cytokine expression and direct cell-to-cell interactions.

This unbiased network analysis of DLB mouse transcriptomes also provides an interesting perspective on α-synuclein related pathology, emphasizing aspects of pathology which can be rescued by NSC transplantation. Networks were enriched in genes associated with mitochondrial and lysosomal function, neurotransmission as well as synaptic plasticity. Mitochondrial dysfunction is prevalent in synucleinopathies as evidenced by clinical findings in patients with PD/DLB who carry genetic mutations of genes *LRRK2* and *PINK1* which are associated with mitochondrial function [38, 53, 100]. However, mitochondrial impairment can also result from α-synuclein over-abundance independently of *LRRK2* or *PINK1* mutations [36, 68, 91]. Our functional annotation implied an over-active state of mitochondrial function in ASO mice characterized by an increase in oxidative stress, apoptosis and DNA damage that was somewhat diminished by NSC transplantation. Similar findings were described in transgenic mice overexpressing the A53T α-synuclein mutation which showed regional associations between mutant protein and neuronal death, dysfunctional mitochondrial protein complex I, increased oxidative stress, and DNA damage [68]. Precisely, how NSC transplantation modulates these systems remains unclear, but these data nevertheless highlight an important new mechanism by which NSC transplantation may influence brain function.

One of the native functions of α-synuclein is the modulation of vesicles at the pre-synaptic terminal, a process that is critical for synaptic signaling [20, 21, 87, 101]. Our network analysis identified several modules associated with changes in dopaminergic, cholinergic, GABAergic, and glutamatergic neurotransmitter systems in response to NSC treatment. Those modules that were enriched in genes related to dopaminergic system also indicated an increased activity in D1 and D2 receptor expressing GABAergic medium spiny neurons as well as modulation in synaptic transmission, postsynaptic density and calcium signaling. These changes in neurotransmitter systems likely relate to our prior findings that mature BDNF produced by NSCs and astrocytes in the striatum is important for NSC-induced behavioral recovery [41]. Binding of BDNF to TrkB receptors which are expressed in striatal medium spiny neurons likely promotes the restoration of normal dendritic morphogenesis. Likewise, binding and subsequent retrograde transport of BDNF/TrkB signaling endosomes in corticostriatal glutamatergic and nigrostriatal dopaminergic projections likely also influences the function and health of substance nigra and cortical neurons that project to the striatum [10, 113].

This analysis uncovered several interesting hub genes in connection to alpha-synuclein pathology and NSC related recovery including *Itpr1*, which has been associated with dopaminergic and Ca^2+^ signaling. This gene encodes a ligand-gated ion channel, an intracellular receptor for inositol 1,4,5-trisphosphate molecules which is highly expressed in neurons [110] and deletions of *Itpr1* are known to cause spinocerebellar ataxia [47]. Furthermore, activation of D1 dopamine receptors within the nucleus accumbens induces Ca^2+^ signals which are critical for neuronal excitability and synaptic plasticity [95]. Thus further investigation of *Itpr1* functions in ASO mice could reveal additional insight about the potential dysregulation of Ca^2+^ homeostasis induced by alpha-synuclein pathology. Another interesting hub gene identified in our analysis is *Elavl4* as several lines of evidence specify roles for this gene in neuronal plasticity, recovery from axonal injury, and learning and memory [84]. In addition, genetic variants in human *ELAVL4* have been associated with age of onset in Parkinson disease (PD) [35]. Besides its role in mRNA stabilization in the brain, the functions of *Elavl4* are still emerging. Therefore, future studies aimed at manipulating *Elavl4* expression in the context of ASO mice and NSC transplantation could uncover important additional roles for this gene in DLB pathogenesis and NSC-mediated behavioral recovery.

## Conclusion

Taken together, our data suggest that NSC transplantation influences multiple gene networks and interacts with endogenous neural and immune cells to improve cognitive and motor behavior in DLB mice. Our analysis greatly extends our prior findings to implicate NSC-induced changes in synaptic plasticity, mitochondrial and lysosomal function, and both innate and adaptive immunity in functional recovery (Additional file 2: Figure S8). It also highlights the potential use of WGCNA analysis to uncover candidate genes such as *Elav﻿l1*and *Itpr1* that may be critically involved in the pathogenesis and/or potential treatment of DLB and warrant further investigation.

## Additional files

Additional file 1: RNA_quality_measurments. Table S2. contains RNA related quality measurements including A260, A280, 260/280, 260/230 and RNA integrity number (RIN). (XLSX 11 kb)
Additional file 2: This file contains six supplemental figures. **Figure S1.** outlines neural stem cell transplantation strategy and WGCNA workflow. **Figure S2.** illustrates result of RNA degradation analysis. **Figure S3.** demonstrates the result of Quality Control (QC) Analysis of gene expression. **Figure S4.** illustrates a dendrogram produced by average linkage hierarchical clustering of approximately 12,00 genes based on the topological overlap matrix (TOM) as input similarities. **Figure S5.** visualizes the association between consensus module eigengenes and quantitative phenotypes related to NSC engraftment. **Figure S6.** depicts a heatmap, visualizing genotype specific gene expression pattern in association to innate immunity. **Figure S7.** shows an enlarged version of Fig. 4D. **Figure S8.** demonstrates a module eigengene (ME) network of 11 significant modules. (PDF 980 kb)
Additional file 3: Codes_and_Parameters. The documents contains codes and parameters used in WGCNA, GSEA and CSEA analysis. (PDF 56 kb)
Additional file 4: GSEA_result. The file contains the result of Gene Set Enrichment Analysis (GSEA) which detects coordinated changes in pre-specified sets of related genes (13 modules) between treatment groups. Each tab of file indicates two conditions in which differential expression of each module as a gene set was tested (tab1: ASO-Veh vs ASO-NSC; tab2: ASO-NSC vs WT-Veh; tab3: WT-NSC vs WT-Veh; tab4 ASO-Veh vs WT-Veh; tab5 ASO-NSC vs WT-NSC). In each tab, first column indicates a module as gene set, the second column indicates the size of the module gene set and the following columns display GSEA statistics. Modules were considered statistically significant at FDR q-val < 0.05. (XLSX 137 kb)
Additional file 5: Pathways_altered_in_ASO_mice_and_NSC_transplantation. Significant differentially regulated pathways between treatment groups summarized in Table S3. The analysis based on GSEA result and CluGo group term cluster annotation which determines the most representative GO term of a cluster of similar biological processes. The first column describes experimental group contrasts, the second columns indicates the significantly differentially expressed module gene sets (Additional file 4) between conditions; the third, fourth and fifth columns contain pathway information of annotated gene sets. Table contains potential dysregulated pathways associated with ASO genotype that are significantly different between ASO-Veh and WT-Veh conditions and pathways that are altered differently in ASO-Veh and ASO-NSC conditions. The overlap of pathway and Module eigegenes direction (Fig. 1, Additional file 4: tab6) between the two contrasts suggest that NSC treatment can alter ASO associated pathways. Pathways that are altered differently in ASO-NSC and WT-NSC conditions suggest an ASO genotype interaction with NSC treatment. (XLSX 359 kb)
Additional file 6: SignificantModules_geneKME_and_phenoCorrelation. Summary output of network analysis result. Each tab of the file is designated to a module. First column of the tab displays gene symbol of genes assigned to the module. Second and third colums indicate color assignment and module number. Fourth column contains intramodular connectivity (KME) (also known as degree) for each gene in the module and the rest of the columns correspond to correlation values of genes for each quantitative phenotypes. (XLSX 725 kb)
Additional file 7: ModuleAnnotation_internalCollection_enrichWGCNA. The file contains the most significant results, description and statistical information of enrichment analyses. Columns are labeled as followed: module number, module color, rank of the significant result, precomputed gene set IDs implemented in WGCNA (dataSetID), data set name (dataSetName), name of gene set library (inGroups), pValue, Bonferroni corrected *p* value, FDR corrected *p* value, the number pf overlapping genes between the gene set and the module (nCommonGenes), ratio of the overlapping genes and the size of module (fracOfEffectiveClassSize), module size (effectiveClassSize), ratio of overlapping genes and the gene set size (fracOfEffectiveSetSize), size of the gene set (effectiveSetSize), name of the gene set (shortDataSetName), symbols of the overlapping genes (overlapGenes). (XLSX 70 kb)
Additional file 8: ModuleAnnotation_CluGo. The result of enrichment analyses performed by CluGO in Cytoscape. ClueGO integrates Gene Ontology (GO) terms as well as pathways and creates a functionally organized GO-pathway term network [14]. Each tab of the file is designated to a module. The columns of each tabs correspond to the significant gene ontology (GO) terms with related single term and group (network) *p*-values in addition to genes associated with GO term. (XLSX 1913 kb)
Additional file 9: ModuleAnnotation_GOenrichmentWGCNA. Supplemantary Gene Ontology Annotation performed in WGCNA. This file contains the first 25 most significant annotation terms for each module based on Gene Ontology gene sets enrichment analysis implemented in WGCNA. Columns in the file are labeled as followed: rank of the significant result (rank), module number (module), module size (modSize), the number of gene from the module that are included in the analysis (bkgrModSize), the size of GO gene set (bkgrTermSize), the number of overlapping genes from the module with GO gene set (nModGenesInTerm), ratio of overlapping gene (nModGenesInTerm) and size of effective module (bkgrModSize), ratio of overlapping gene nModGenesInTerm) and size of GO gene set, enrichment *P* value, Bonferroni corrected P, GO ID (termID), GO term, GO term name and GO term definition. (XLSX 81 kb)
Additional file 10: ModuleAnnotation_enrichR_M1. Annotation result of M1 performed in enrichR. EnrichR currently contains annotated gene sets from 102 gene set libraries organized in 8 categories. Details of the gene set libraries in EnrichrR can be found in publications [25, 56]. Each tab of the file contains gene sets of a particular library that was found to be enriched in the module. The first column represent the gene set (Term), the second column shows the ratio of overlapping module genes with the gene set (Overlap), the following four columns are related to statistics (*P*-value, Adjusted *P*-value, Z-score, Combined Score) and the fifth column shows the symbols of the overlapping genes. The gene sets are ranked by combined score. (XLSX 2936 kb)
Additional file 11: ModuleAnnotation_enrichR_M2. Annotation result of M2 performed in enrichR. EnrichR currently contains annotated gene sets from 102 gene set libraries organized in 8 categories. Details of the gene set libraries in EnrichrR can be found in publications [25, 56]. Each tab of the file contains gene sets of a particular library that was found to be enriched in the module. The first column represent the gene set (Term), the second column shows the ratio of overlapping module genes with the gene set (Overlap), the following four columns are related to statistics (*P*-value, Adjusted *P*-value, Z-score, Combined Score) and the fifth column shows the symbols of the overlapping genes. The gene sets are ranked by combined score. (XLSX 822 kb)
Additional file 12: ModuleAnnotation_enrichR_M3. Annotation result of M3 performed in enrichR. EnrichR currently contains annotated gene sets from 102 gene set libraries organized in 8 categories. Details of the gene set libraries in EnrichrR can be found in publications. Each tab of the file contains gene sets of a particular library that was found to be enriched in the module. The first column represent the gene set (Term), the second column shows the ratio of overlapping module genes with the gene set (Overlap), the following four columns are related to statistics (*P*-value, Adjusted *P*-value, Z-score, Combined Score) and the fifth column shows the symbols of the overlapping genes. The gene sets are ranked by combined score. (XLSX 1032 kb)
Additional file 13: ModuleAnnotation_enrichR_M4. Annotation result of M4 performed in enrichR. EnrichR currently contains annotated gene sets from 102 gene set libraries organized in 8 categories. Details of the gene set libraries in EnrichrR can be found in publications [25, 56]. Each tab of the file contains gene sets of a particular library that was found to be enriched in the module. The first column represent the gene set (Term), the second column shows the ratio of overlapping module genes with the gene set (Overlap), the following four columns are related to statistics (*P*-value, Adjusted *P*-value, Z-score, Combined Score) and the fifth column shows the symbols of the overlapping genes. The gene sets are ranked by combined score. (XLSX 671 kb)
Additional file 14: ModuleAnnotation_enrichR_M10. Annotation result of M10 performed in enrichR. EnrichR currently contains annotated gene sets from 102 gene set libraries organized in 8 categories. Details of the gene set libraries in EnrichrR can be found in publications [25, 56]. Each tab of the file contains gene sets of a particular library that was found to be enriched in the module. The first column represent the gene set (Term), the second column shows the ratio of overlapping module genes with the gene set (Overlap), the following four columns are related to statistics (*P*-value, Adjusted *P*-value, Z-score, Combined Score) and the fifth column shows the symbols of the overlapping genes. The gene sets are ranked by combined score. (XLSX 350 kb)
Additional file 15: ModuleAnnotation_enrichR_M11. Annotation result of M11 performed in enrichR. Each tab of the file is the outcome of a gene set library enrichment analysis implemented in enrichR. (XLSX 433 kb)
Additional file 16: ModuleAnnotation_enrichR_M13. Annotation result of M13 performed in enrichR. EnrichR currently contains annotated gene sets from 102 gene set libraries organized in 8 categories. Details of the gene set libraries in EnrichrR can be found in publication [25, 56]. Each tab of the file contains gene sets of a particular library that was found to be enriched in the module. The first column represent the gene set (Term), the second column shows the ratio of overlapping module genes with the gene set (Overlap), the following four columns are related to statistics (*P*-value, Adjusted *P*-value, Z-score, Combined Score) and the fifth column shows the symbols of the overlapping genes. The gene sets are ranked by combined score. (XLSX 216 kb)
Additional file 17: ModuleAnnotation_enrichR_M15. Annotation result of M15 performed in enrichR. EnrichR currently contains annotated gene sets from 102 gene set libraries organized in 8 categories. Details of the gene set libraries in EnrichrR can be found in publications [25, 56]. Each tab of the file contains gene sets of a particular library that was found to be enriched in the module. The first column represent the gene set (Term), the second column shows the ratio of overlapping module genes with the gene set (Overlap), the following four columns are related to statistics (*P*-value, Adjusted *P*-value, Z-score, Combined Score) and the fifth column shows the symbols of the overlapping genes. The gene sets are ranked by combined score. (XLSX 431 kb)
Additional file 18: ModuleAnnotation_enrichR_M16. Annotation result of M16 performed in enrichR. EnrichR currently contains annotated gene sets from 102 gene set libraries organized in 8 categories. Details of the gene set libraries in EnrichrR can be found in publications [25, 56]. Each tab of the file contains gene sets of a particular library that was found to be enriched in the module. The first column represent the gene set (Term), the second column shows the ratio of overlapping module genes with the gene set (Overlap), the following four columns are related to statistics (*P*-value, Adjusted *P*-value, Z-score, Combined Score) and the fifth column shows the symbols of the overlapping genes. The gene sets are ranked by combined score. (XLSX 125 kb)
Additional file 19: ModuleAnnotation_enrichR_M17. Annotation result of M17 performed in enrichR. EnrichR currently contains annotated gene sets from 102 gene set libraries organized in 8 categories. Details of the gene set libraries in EnrichrR can be found in publications [25, 56]. Each tab of the file contains gene sets of a particular library that was found to be enriched in the module. The first column represent the gene set (Term), the second column shows the ratio of overlapping module genes with the gene set (Overlap), the following four columns are related to statistics (*P*-value, Adjusted *P*-value, Z-score, Combined Score) and the fifth column shows the symbols of the overlapping genes. The gene sets are ranked by combined score. (XLSX 412 kb)
Additional file 20: ModuleAnnotation_enrichR_M18. Annotation result of M18 performed in enrichR. EnrichR currently contains annotated gene sets from 102 gene set libraries organized in 8 categories. Details of the gene set libraries in EnrichrR can be found in publications [25, 56]. Each tab of the file contains gene sets of a particular library that was found to be enriched in the module. The first column represent the gene set (Term), the second column shows the ratio of overlapping module genes with the gene set (Overlap), the following four columns are related to statistics (*P*-value, Adjusted *P*-value, Z-score, Combined Score) and the fifth column shows the symbols of the overlapping genes. The gene sets are ranked by combined score. (XLSX 72 kb)

## Abbreviations

ASO: Overexpressed wild-type human α-synuclein
BDNF: Brain–derived neurotrophic factor
BT: Beam Transversal
DLB: Dementia with Lewy Body
GLT-1: Glutamate type I transporter
NO: Novel Object
NP: Novel Place
NSCs: Neural stem cells
WGCNA: Weighted co-expression network analysis
