# Supplementary material for: Integrated analysis of genetic, behavioral, and biochemical data implicates neural stem cell-induced changes in immunity, neurotransmission and mitochondrial function in Dementia with Lewy Body mice
Source: Acta Neuropathol Commun. 2017 Mar 10;5:21. doi: 10.1186/s40478-017-0421-0 (PMC5345195; doi:10.1186/s40478-017-0421-0)
Supplement: Additional file 2: — This file contains six supplemental figures. Figure S1. outlines neural stem cell transplantation strategy and WGCNA workflow. Figure S2. illustrates result of RNA degradation analysis. Figure S3. demonstrates the result of Quality Control (QC) Analysis of gene expression. Figure S4. illustrates a dendrogram produced by average linkage hierarchical clustering of approximately 12,00 genes based on the topological overlap matrix (TOM) as input similarities. Figure S5. visualizes the association between consensus module eigengenes and quantitative phenotypes related to NSC engraftment. Figure S6. depicts a heatmap, visualizing genotype specific gene expression pattern in association to innate immunity. Figure S7. shows an enlarged version of Fig. 4D. Figure S8. demonstrates a module eigengene (ME) network of 11 significant modules. (PDF 980 kb) [file 40478_2017_421_MOESM2_ESM.pdf]

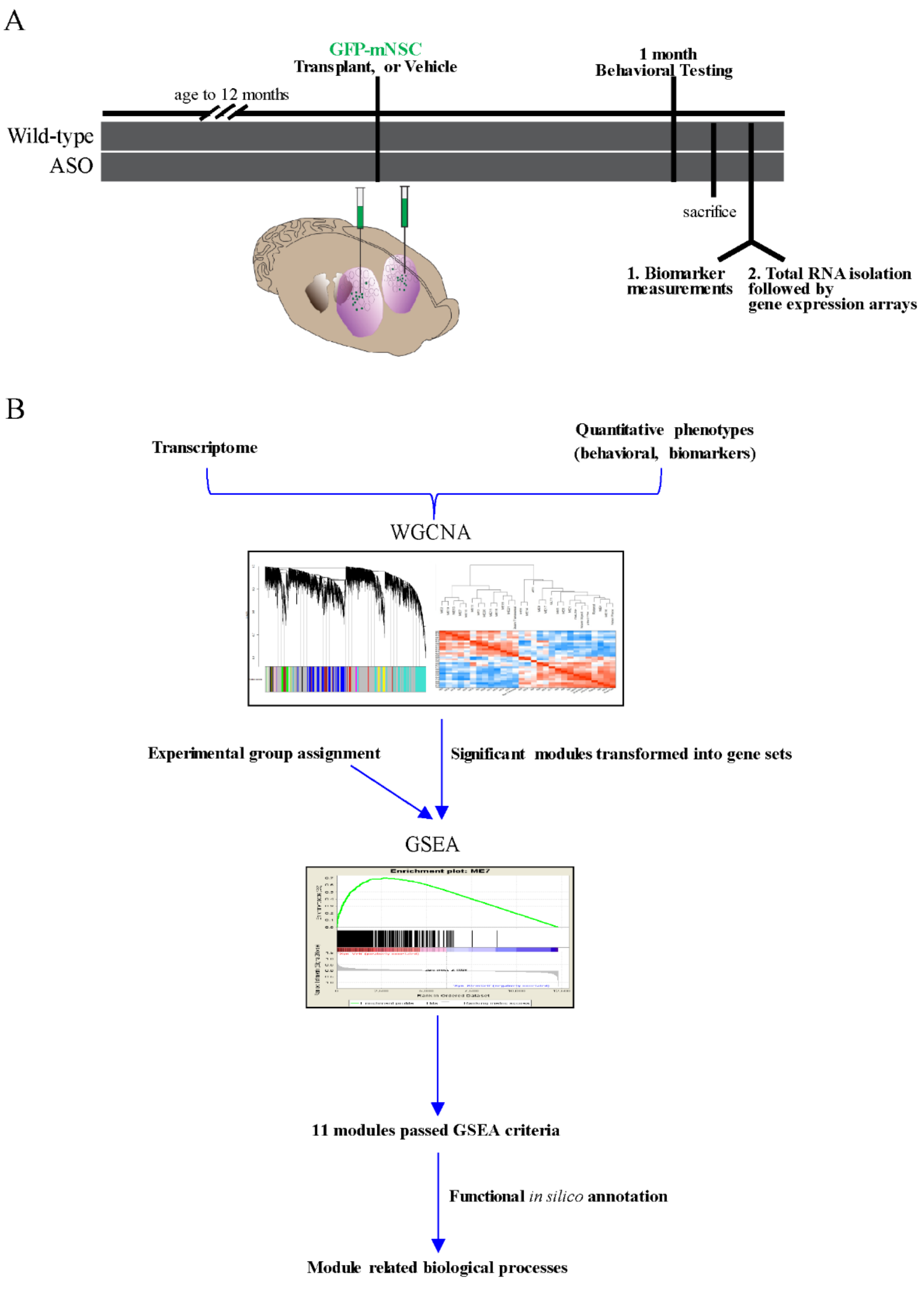

A
GFP-mNSC
Transplant, or Vehicle
1 month
Behavioral Testing
age to 12 months
Wild-type
ASO
sacrifice
1. Biomarker measurements
2. Total RNA isolation followed by gene expression arrays
B
Transcriptome
Quantitative phenotypes (behavioral, biomarkers)
WGCNA
Experimental group assignment
Significant modules transformed into gene sets
GSEA
Enrichment plot: ME7
11 modules passed GSEA criteria
Functional in silico annotation
Module related biological processes

**Figure S1. Neural stem cell transplantation strategy and WGCNA workflow**. **(A)** Hippocampal/cortical NSCs derived from syngeneic GFP-transgenic mice at postnatal day 1 (P1) were grown as adherent monolayers and transplanted bilaterally into the dorsal striatum. Behavioral studies were performed beginning 1 month after transplantation. Motor function was tested by Rotarod pole and Beam Transversal tests, whereas cortical- and hippocampal-dependent learning and memory were examined using Novel Object and Novel Place Recognition paradigms. Mice were then sacrificed and striata microdissected for further assessment. Total RNA was isolated to assess gene expression and Western blots performed to measure BDNF, pSer31TH and GLT1 levels. **(B)** Striatal transcriptomes were integrated with the quantitative behavioral and biomarker phenotypes in WGCNA, identifying significant association between gene networks (modules) and phenotypes. Significant networks were combined with gene expression levels across experimental groups in GSEA to assess gene enrichment changes. Modules showing significant enrichment in ASO-Veh versus ASO-Veh comparison were subjected to *in silico* functional annotation.

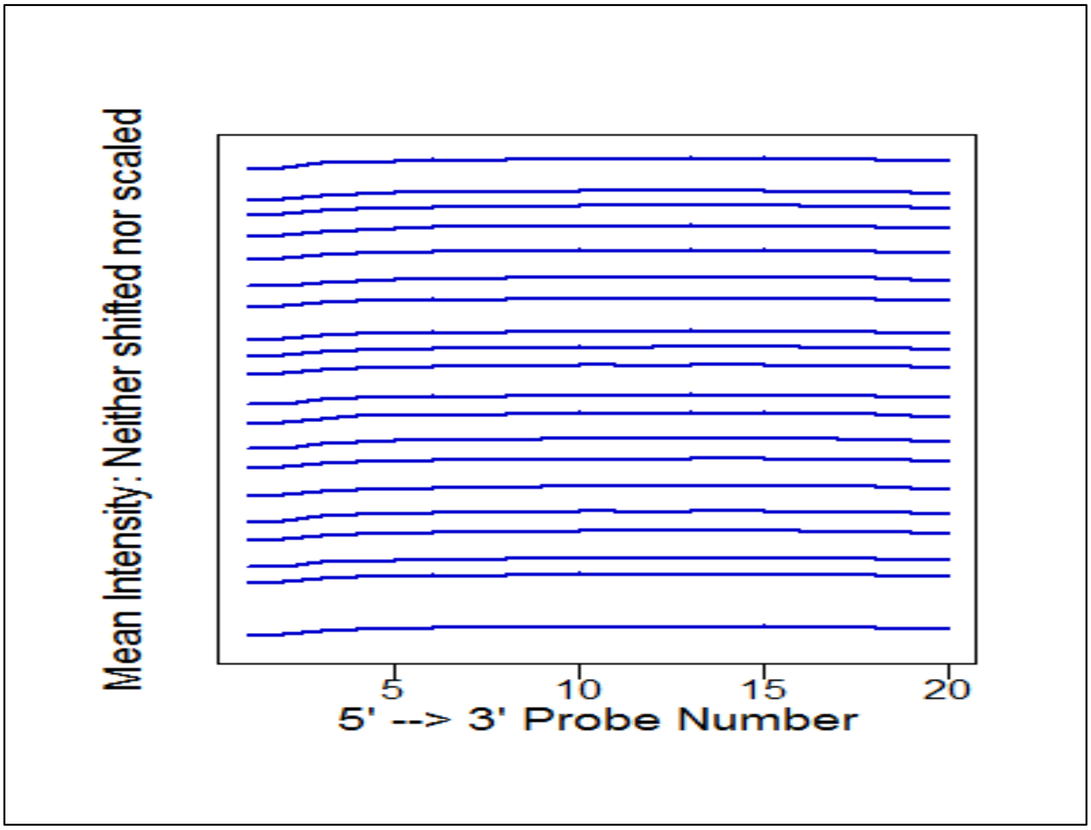


**Figure S2. RNA degradation plot.** The Affymetrix Mouse Gene 2.0 ST Array is designed with a median of 21 unique probes per transcript. Each unique probe is 25 bases in length measuring a median of 525 bases per transcript. Each line on the plot represents an individual sample (n=20). Degradation lines do not show substantially lower intensity at the 5' end relative to 3', therefore RNA quality is high and suggests no degradation in concordance with the reported RIN > 9 numbers.

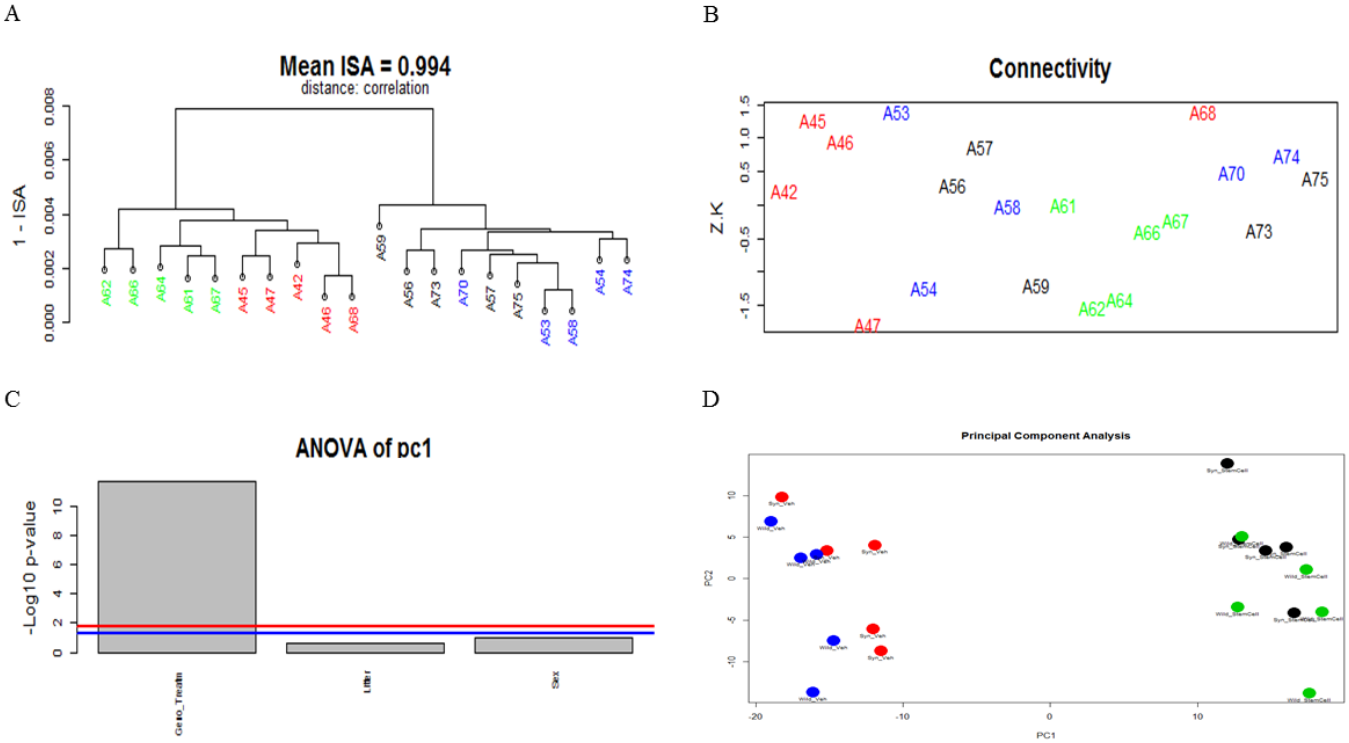


**Figure S3. Quality Control (QC) Analysis.** All transcriptome samples were subject to quantitative control (QC) analysis preceding network construction to detect any potential confounding effects and outliers. All samples were controlled for litter and sex by SampleNetwork implemented in ampleNetwork1.07 (Oldham et al 2012). **(A)** Sample relationships are depicted in a dendrogram with respect to a correlation matrix. Samples are clustered on the distance of mean intersample adjacency (ISA, or density). All samples cluster together tightly except A59 which is designated as outlier and left out from the association analysis. Samples are colored with respect to their group assignment. **(B)** An additional measurement of sample relationships uses the network concept of standardized connectivity. Standardized connectivity (*Z.K*) describes the overall strength of connections between a node and all of the other nodes in the network. Samples with lower than -2.5 of Z.K. are considered outliers. **(C)** Multivariate linear regression revealed a highly significant effect of genotype-treatment (Geno_Treat) on the first principal component 1 (PC1) of the entire network (Blue line: $P = .05$; red line: Bonferroni correction for multiple comparisons). **(D)** A principal component analysis (PCA) distributes samples based on PC1 and principal component 2 (PC2) expression. The separation between groups suggests a strong effect of stem cell engrafting on gene expression. Samples are colored with respect to their group assignment (green:WT-NSC; red:ASO-Veh; blue:WT-Veh, black:ASO-NSC).

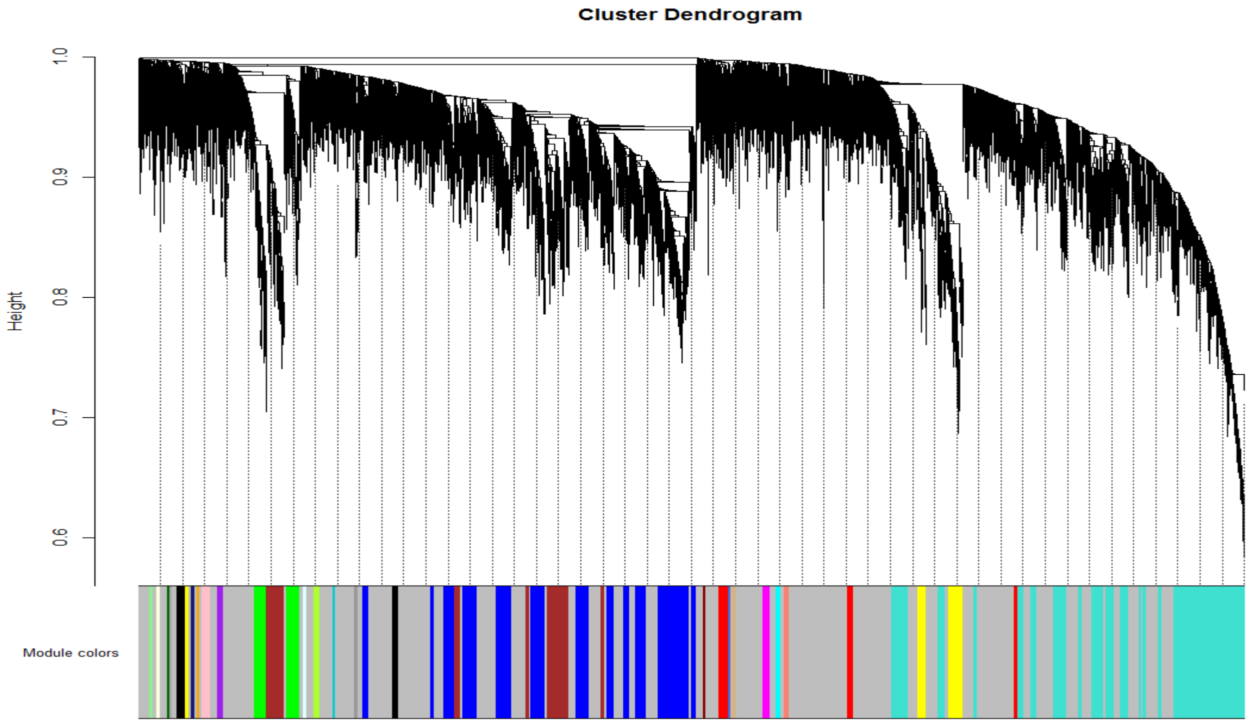


**Figure S4. WGCNA correlation network module clusters.** Approximately 12,00 genes in the mouse striatum cluster based on topological overlap (TO). Tightly correlated sets of genes are clustered using “biweight midcorrelation” (also called bicor) and “signed network” function implemented in WGCNA. All possible pairwise correlations are converted into measures of connection strength by taking their absolute values and raising them to a power of 20 (β=20). The color bands provide a simple visual of module assignments representing a module.

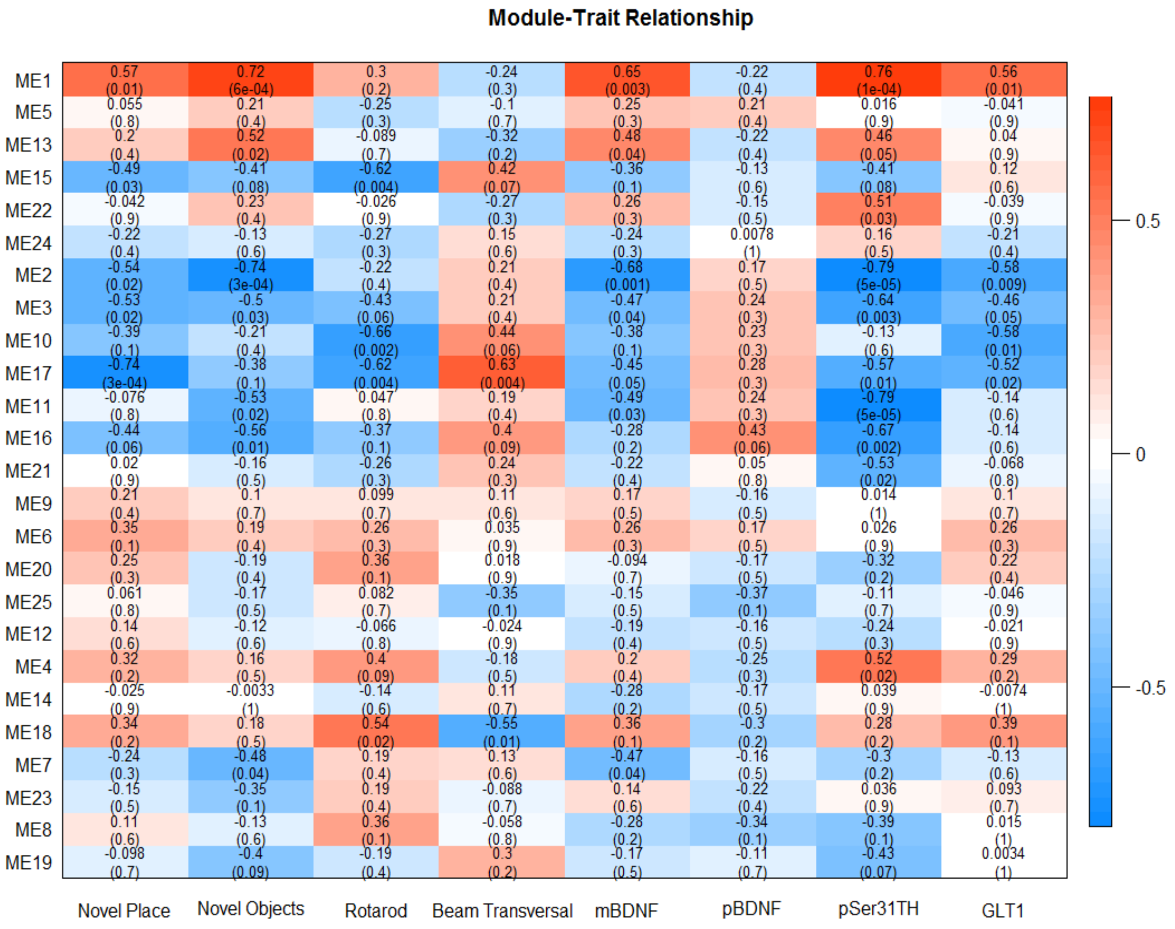


**Figure S5**. **Relationship of consensus module eigengenes and quantitative phenotypes related to NSC engraftment**. Each row in the table corresponds to a consensus module, and each column to a quantitative phenotype. Numbers in the table show the correlation values (r) of the corresponding module eigengenes and phenotypes with the p-values printed below in parentheses. The table is color coded by correlation strength and direction according to the color legend. Red represents a positive correlation and blue represent a negative correlation between module and phenotype.

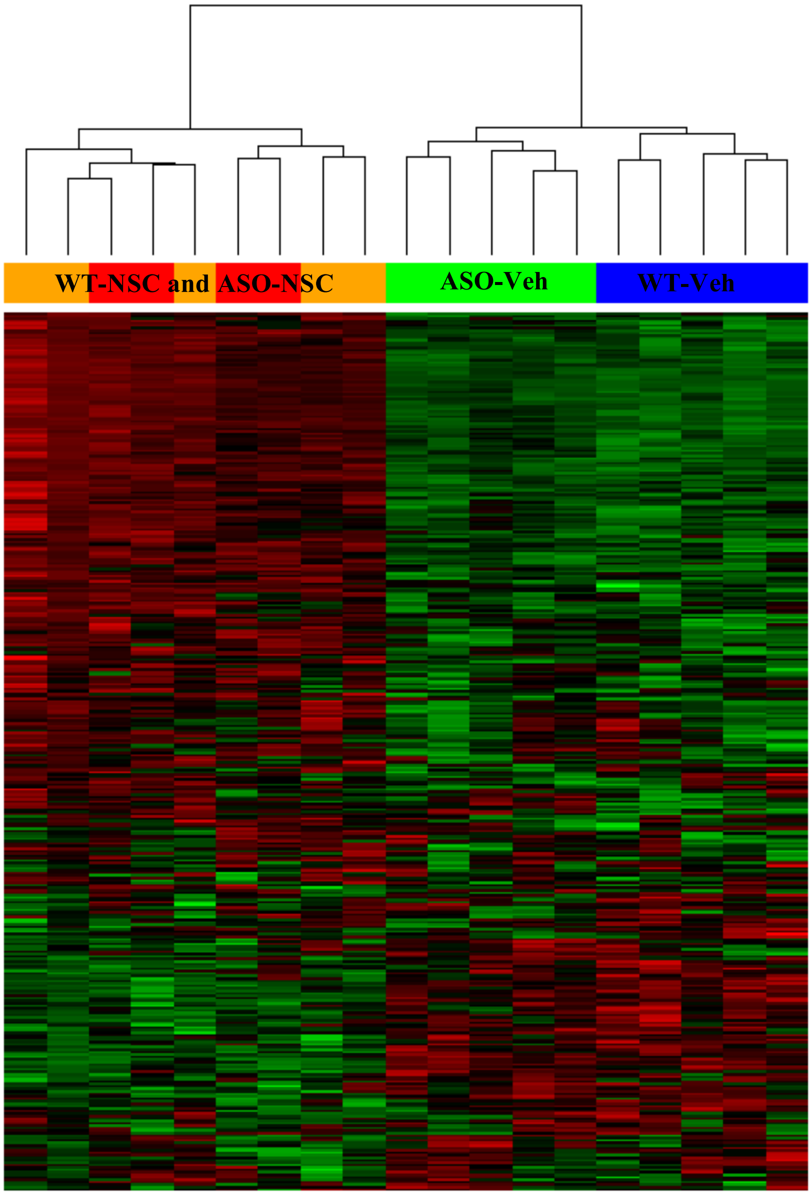


**Figure S6. Heatmap visualization of gene expression associated with Innate Immunity.** Genes identified as part of innate immunity (InnateDB, http://www.innatedb.com) were subjected to unsupervised clustering. The clustering revealed a distinct separation between WT-Veh (blue) and ASO-Veh mice (light green) and an overall grouping of all stem cell treated animals regardless of genotype (WT-NSC:orange) and ASO-NSC:red)

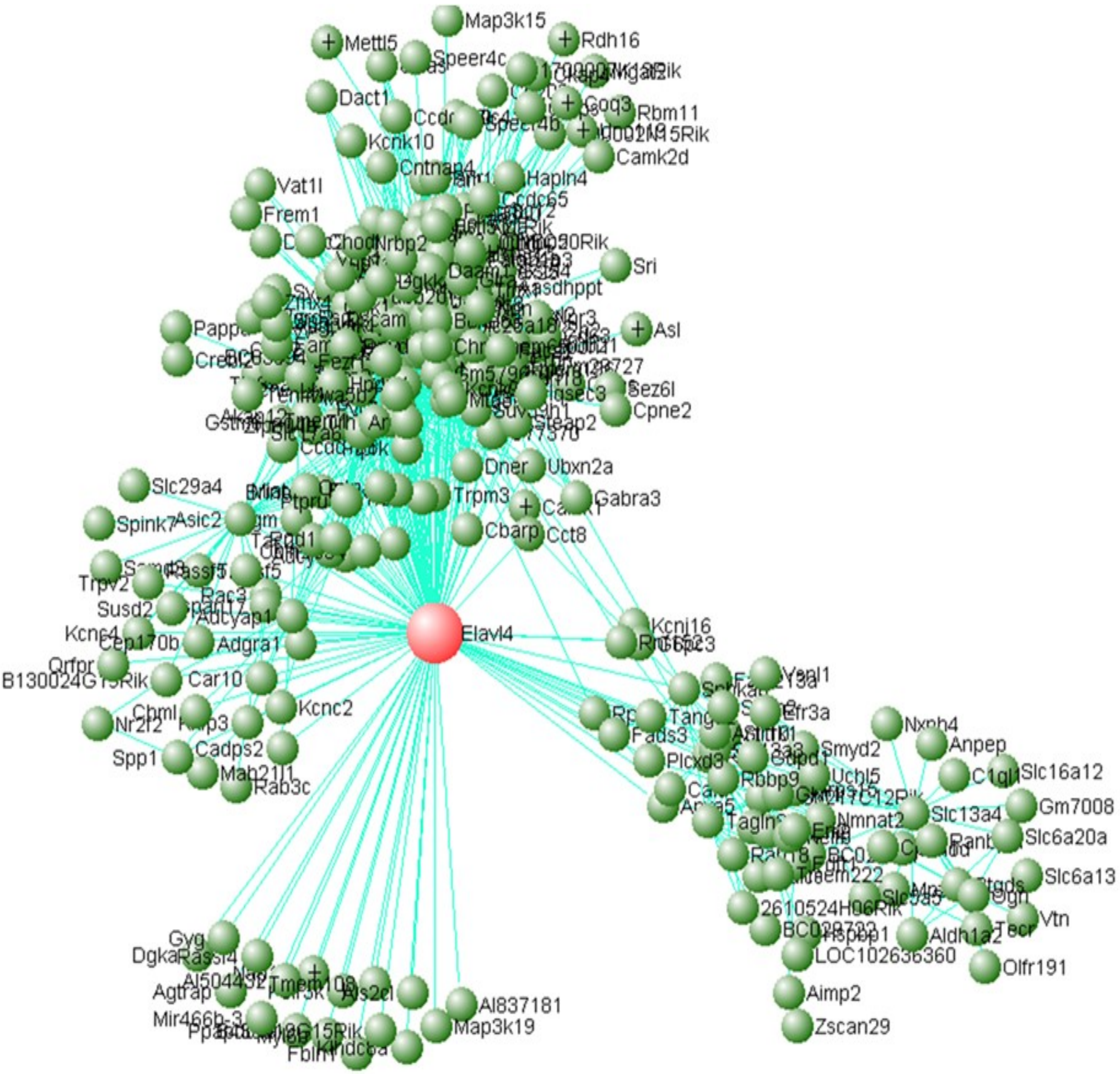


**Figure S7. The modular network organization of M3 depicted by VisANT** [120]. Each green node represents a unique gene in the M3 module with the gene-gene interactions depicted in cyan lines. The red center node corresponds to the module hubgene, Elavl4, with highest intramodular connectivity holding together the entire network as a functional unit.

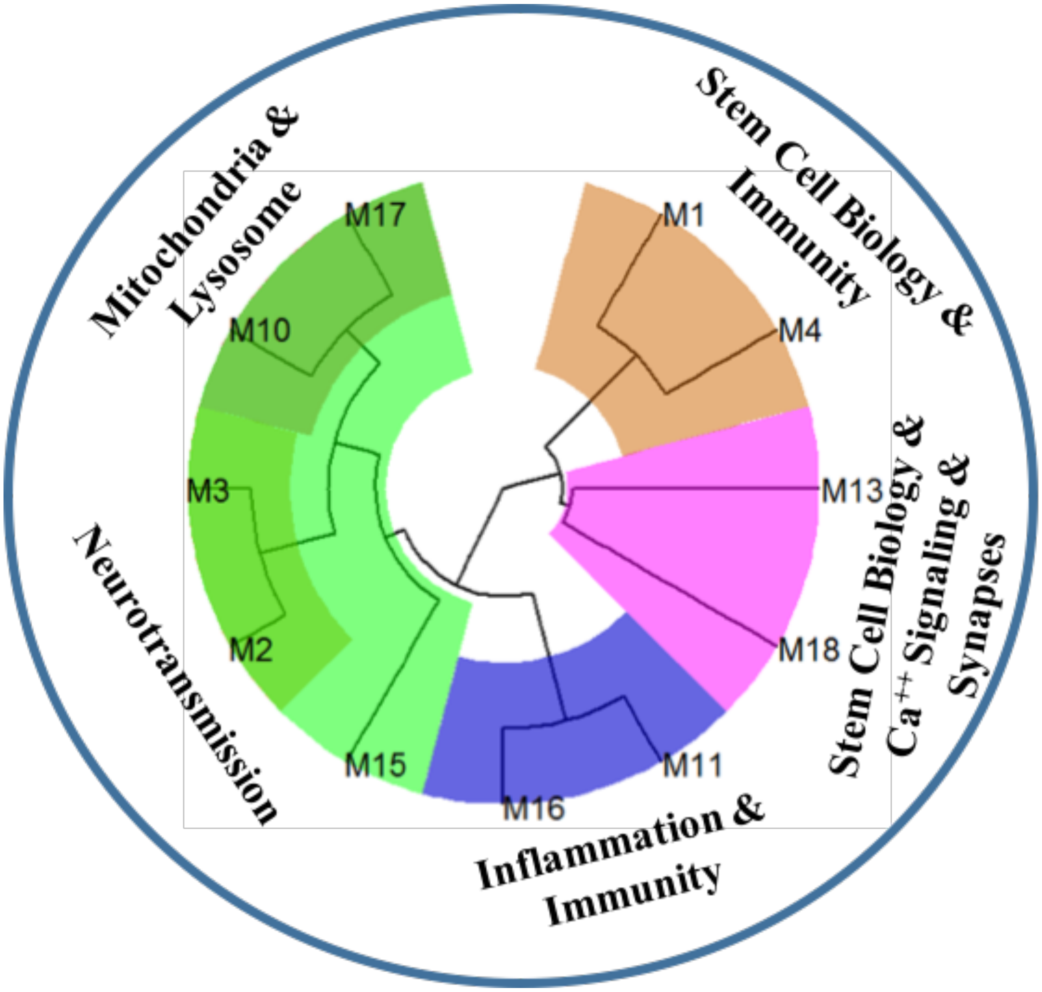


**Figure S8. Module EigenGene (ME) Network.** The overall ME network reveals modules with similar expression patterns as well as related functional categories. A broader functional annotation of M1 and M4 is related to stem cell biology, and inflammation and immunity. M13 and M18 highlights potential biological processes related to stem cell biology and synaptic activity. M11 and M16 are rather associated to a modulated inflammatory state. M2 and M3 underscore the multiple neurotamitter systems in striatum. M10 and M17 implicate mitochondrial and lysosomal related biological processes.
