# Supplementary material for: Integrated analysis of genetic, behavioral, and biochemical data implicates neural stem cell-induced changes in immunity, neurotransmission and mitochondrial function in Dementia with Lewy Body mice
Source: Acta Neuropathol Commun. 2017 Mar 10;5:21. doi: 10.1186/s40478-017-0421-0 (PMC5345195; doi:10.1186/s40478-017-0421-0)
Supplement: Additional file 3: — Codes_and_Parameters. The documents contains codes and parameters used in WGCNA, GSEA and CSEA analysis. (PDF 56 kb) [file 40478_2017_421_MOESM3_ESM.pdf]

```
###############################################################################
##########                        WGCNA                             ##########
###############################################################################
#Probe filter
nsFilter(eset, require.entrez=TRUE, remove.dupEntrez=TRUE,
var.filter=TRUE, var.cutoff=0.5, feature.exclude="^AFFX")$eset

#Outlier detection
A=adjacency(t(datExpr), type="signed", corFnc = "bicor")
heatmap(A, labRow = pData(eset)$Genotype)
k=as.numeric(apply(A,2,sum))-1
Z.k=scale(k)
thresholdZ.k=-2.5
outlierColor=ifelse(Z.k<thresholdZ.k,"blue","black")

#Power determination
sft = pickSoftThreshold(datExpr, dataIsExpr = TRUE,powerVector = powers,
networkType = "signed" , verbose = 5, corOptions =list(use='p'),  corFnc
= bicor)

sft$powerEstimate

plot(sft$fitIndices[,1],  -sign(sft$fitIndices[,3])*sft$fitIndices[,2],
xlab="Soft Threshold (power)",ylab="Scale Free Topology Model Fit,
signed R^2",type="n",main = paste("Scale independence"));
text(sft$fitIndices[,1],                                               -
sign(sft$fitIndices[,3])*sft$fitIndices[,2],labels=powers,cex=cex1,col
="red");
abline(h=0.80, col="red")

#Mean connectivity as a function of the soft-thresholding power
plot(sft$fitIndices[,1],   sft$fitIndices[,5],   xlab="Soft   Threshold
(power)",ylab="Mean  Connectivity,  signed  R^2",  type="n",  main  =
paste("Mean connectivity"))
text(sft$fitIndices[,1],sft$fitIndices[,5],labels=powers,cex=cex1,col=
"red")

#Module Construction
net  <-  blockwiseModules(t(datExpr),  power=20,  numericLabels=TRUE,
deepSplit=4,
minModuleSize=30,minKMEtoStay=0,mergeCutHeight=0.25,detectCutHeight=0.
99995,corType="bicor", networkType="signed", pamStage=FALSE, verbose=3,
saveTOMs=TRUE,maxBlockSize=15000, randomSeed = 12345)
ls(net)
table(net$colors)
moduleLabels = net$colors
```

```
mergedColors = labels2colors(net$colors)
moduleColors = labels2colors(net$colors)
#par(mfrow=c(4,2))

plotDendroAndColors(net$dendrograms[[1]],
mergedColors[net$blockGenes[[1]]], "Module colors", dendroLabels =
FALSE, hang = 0.03, addGuide = TRUE, guideHang = 0.05)
```

##
##########   GSEA   ##########
##

We used a standalone version of GSEA developed by the Broad Institute (http://software.broadinstitute.org/gsea/) [1, 2]. The parameters were chosen following the user guide (http://software.broadinstitute.org/gsea/doc/GSEAUserGuideFrame.html)

The following parameters were used in our experiment:

```
producer_class	xtools.gsea.Gsea
producer_timestamp 1487519154693
param collapse	false
param cls
	C:\reasult_2016\GSEA\pheno_wildveh_synstemcell.cls#Syn_StemCell_versus_Wild_Veh
param plot_top_x 20
param norm	meandiv
param save_rnd_lists	false
param median	false
param num	100
param scoring_scheme	weighted
param make_sets	true
param mode	Max_probe
param gmx	C:\reasult_2016\rediced_dataset\GSEA\GENESET_13_nodes.gmx
param gui	false
param chip
	ftp://gseaftp.broadinstitute.org/pub/gsea/annotations/GENE_SYMBOL.chip
param metric	Diff_of_Classes
param rpt_label	ASO_NSC_vs_WT-TVeh
param help	false
param order descending
param out	C:\reasult_2016\GSEA
param create_svgs	false
param permute	gene_set
param rnd_type	no_balance
param set_min	15
param include_only_symbols	true
```

```
param sort  real
param create_gcts      false
param rnd_seed   timestamp
param nperm 1000
param zip_report false
param set_max    50000
param res   C:\reasult_2016\GSEA\exprs_wildveh_synstemcell.gct
```

[1] A. Subramanian, H. Kuehn, J. Gould, P. Tamayo, J.P. Mesirov, GSEA-P: a desktop application for Gene Set Enrichment Analysis, Bioinformatics 23(23) (2007) 3251-3.
[2] A. Subramanian, P. Tamayo, V.K. Mootha, S. Mukherjee, B.L. Ebert, M.A. Gillette, A. Paulovich, S.L. Pomeroy, T.R. Golub, E.S. Lander, J.P. Mesirov, Gene set enrichment analysis: a knowledge-based approach for interpreting genome-wide expression profiles, Proc Natl Acad Sci U S A 102(43) (2005) 15545-50.

##
##########   CSEA   ##########
##

This tool is an online application with fixed parameters (http://genetics.wustl.edu/jdlab/csea-tool-2/).

The description of the tool and the statistical model implemented in SEA is published in [3, 4]

[3] X. Xu, A.B. Wells, D.R. O'Brien, A. Nehorai, J.D. Dougherty, Cell type-specific expression analysis to identify putative cellular mechanisms for neurogenetic disorders, J Neurosci 34(4) (2014) 1420-31.
[4] J.D. Dougherty, E.F. Schmidt, M. Nakajima, N. Heintz, Analytical approaches to RNA profiling data for the identification of genes enriched in specific cells, Nucleic Acids Res 38(13) (2010) 4218-30.
